# Missing value replacement in strings and applications

**DOI:** 10.1007/s10618-024-01074-3

**Published:** 2025-01-22

**Authors:** Giulia Bernardini, Chang Liu, Grigorios Loukides, Alberto Marchetti-Spaccamela, Solon P. Pissis, Leen Stougie, Michelle Sweering

**Affiliations:** 1 Department of Mathematics, Informatics and Geosciences, University of Trieste, Trieste, Italy; 2 Medical Center, Zhejiang University, Zhejiang, China; 3 Department of Informatics, King’s College London, London, UK; 4 Department of Computer, Automatic and Management Engineering, La Sapienza University of Rome, Rome, Italy; 5 CWI, Amsterdam, The Netherlands; 6 Faculty of Science, Vrije Universiteit, Amsterdam, The Netherlands; 7 ERABLE Team, Lyon, France; 8 School of Business and Economics, Vrije Universiteit, Amsterdam, The Netherlands

**Keywords:** String algorithms, Forbidden patterns, Missing value replacement, String sanitization

## Abstract

Missing values arise routinely in real-world sequential (string) datasets due to: (1) imprecise data measurements; (2) flexible sequence modeling, such as binding profiles of molecular sequences; or (3) the existence of confidential information in a dataset which has been deleted deliberately for privacy protection. In order to analyze such datasets, it is often important to replace each missing value, with one or more *valid* letters, in an efficient and effective way. Here we formalize this task as a combinatorial optimization problem: the set of constraints includes the *context* of the missing value (i.e., its vicinity) as well as a finite set of user-defined *forbidden* patterns, modeling, for instance, implausible or confidential patterns; and the objective function seeks to *minimize the number of new letters* we introduce. Algorithmically, our problem translates to finding shortest paths in special graphs that contain *forbidden edges* representing the forbidden patterns. Our work makes the following contributions: (1) we design a linear-time algorithm to solve this problem for strings over constant-sized alphabets; (2) we show how our algorithm can be effortlessly applied to *fully* sanitize a private string in the presence of a set of fixed-length forbidden patterns [Bernardini et al. 2021a]; (3) we propose a methodology for sanitizing and clustering a collection of private strings that utilizes our algorithm and an effective and efficiently computable distance measure; and (4) we present extensive experimental results showing that our methodology can efficiently sanitize a collection of private strings while preserving clustering quality, outperforming the state of the art and baselines. To arrive at our theoretical results, we employ techniques from formal languages and combinatorial pattern matching.

## Introduction

A string is a sequence of letters over some alphabet. Strings are one of the most fundamental data types. They are used to model, among others, genetic information, with letters representing DNA bases (Koboldt et al 2013), location information, with letters representing check-in’s of individuals at different locations (Ying et al 2011), or natural language information, with letters representing words in some document (Aggarwal and Zhai 2012).

Missing values arise routinely in real-world sequential datasets:

1. Due to imprecise, incomplete or unreliable data measurements, such as streams of sensor measurements, RFID measurements, trajectory measurements, or DNA sequencing reads (Rubin and Little 2019; Aggarwal 2009; Aggarwal and Yu 2009; Li et al 2009; Li and Durbin 2010).
2. Due to (deliberate) flexible sequence modeling, such as binding profiles of molecular sequences (Staden 1984; Alzamel et al 2020; Wuilmart et al 1977).
3. When strings contain confidential information (patterns) which has been deleted deliberately for privacy protection (Aggarwal 2008; Bernardini et al 2021a; Mieno et al 2021).

Let us denote by *w* the input string, by $\Sigma$ the original alphabet, and by $\#\notin \Sigma$ the letter representing a missing value in *w*. It is often important to be able to replace each missing value in *w* with one or more *valid* letters (letters from $\Sigma$) efficiently and effectively. Let us give a few examples:

1. In bioinformatics, since the DNA alphabet consists of four letters, i.e., $\Sigma =\{\texttt {A}, \texttt {C}, \texttt {G}, \texttt {T}\}$, many off-the-shelf algorithms for processing DNA data use a two-bits-per-base encoding to compactly represent the DNA alphabet ($\texttt {A}\iff 00, \texttt {C}\iff 01, \texttt {G}\iff 10, \texttt {T}\iff 11$). In order to use these algorithms when *w* contains unknown bases ($\#$ letters), we would have to modify these algorithms to work on the extended alphabet $\{\texttt {A}, \texttt {C}, \texttt {G}, \texttt {T}, \texttt {\#}\}$. This solution may have a negative impact on the time efficiency of the algorithms or the space efficiency of the data structures they use. Thus, instead, in several state-of-the-art DNA data processing tools (e.g., (Li et al 2009; Li and Durbin 2010)), the occurrences of $\#$ are replaced by a fixed or random letter from $\{\texttt {A}, \texttt {C}, \texttt {G}, \texttt {T}\}$, so that off-the-shelf algorithms can be directly used. This, however, may introduce spurious patterns, including patterns that are unlikely to occur in a DNA sequence (Brendel et al 1986; Régnier and Vandenbogaert 2006).
2. In data sanitization, the occurrences of $\#$ in *w* may reveal the *locations* of sensitive patterns modeling confidential information (Bernardini et al 2021a). Thus, an adversary who knows *w*, the dictionary of sensitive patterns, and how $\#$’s have been added in *w* may infer a sensitive pattern. To prevent this, the occurrences of $\#$’s must be ultimately replaced by letters of $\Sigma$. This replacement gives rise to another string *y* over $\Sigma$ which must ensure that sensitive patterns, as well as any implausible patterns (i.e., known or likely artefacts of sanitization that could be exploited to locate the positions of replaced $\#$’s) do not occur in *y*.
3. In databases, some values may be missing because of errors due to system failures, when data are collected automatically, or because of users’ unwillingness to provide these values, due to privacy concerns (Aggarwal and Parthasarathy 2001). Replacing missing values is important to improve the quality of query answers (Bießmann et al 2018) or to build accurate models from data (Dong et al 2015). Meanwhile, there are dependencies among values leading to constraints which must be satisfied by missing value replacement methods, such as integrity constraints (Rekatsinas et al 2017) and functional dependencies (Breve et al 2022).

The aforementioned constraints motivate us to formalize the task of missing value replacement in strings as a combinatorial optimization problem, which we call the Missing Value Replacement in Strings problem (MVRS, in short). The set of constraints of MVRS includes: (1) the *context* of the missing value (i.e., its vicinity, which is important for the sequential structure of the string); and (2) a finite set of user-defined *forbidden* patterns (i.e., patterns which should not be introduced as a byproduct of the replacement). The objective function of MVRS seeks to minimize the number of added letters. In particular, minimizing the number of added letters implies that the *k-gram distance* (Ukkonen 1992) between the input and the output string is minimized.^1^ Let $\Sigma ^*$ denote the set of all strings over $\Sigma$. The MVRS problem is defined as follows.

**Table:** 

Missing Value Replacement in Strings (MVRS)	
**Input:** Two strings $u,v\in \Sigma ^{*}$ and a finite set $S\subset \Sigma ^{*}$.	
**Output:** A shortest string $x\in \Sigma ^{*}$ such that *u* is a prefix of *x*, *v* is a suffix of *x*, and no $s\in S$ occurs in *x*; or FAIL if no such *x* exists.	

Let us now directly link the definition of MVRS to the context of missing value replacement. String *u* is the *left context*; i.e., a string of arbitrary length that occurs right *before* the missing value. String *v* is, analogously, the *right context*; i.e., a string of arbitrary length that occurs right *after* the missing value. The missing value thus lies in between *u* and *v*. The finite set *S* corresponds to the set of forbidden patterns. The output string *x* corresponds to a string that could be used to replace $u \# v$, where $\#$ denotes a missing value. Finally, minimizing the length of the output string *x* corresponds to the fact that we want to keep the output string as similar as possible to $u \# v$ by introducing the *smallest number of new letters*. It should now be clear that a *single* instance of MVRS corresponds to *one* missing value replacement.

We now consider the simple case in which all forbidden patterns have fixed length *k*. In this case, MVRS translates to a *reachability* problem in de Bruijn graphs: we seek for a shortest path in the complete de Bruijn graph of order *k* over $\Sigma$ (de Bruijn 1946) in the presence of forbidden edges.^2^ Let $\Sigma ^k$ be the set of all length-*k* strings over $\Sigma$. The *complete de Bruijn graph* of order *k* over an alphabet $\Sigma$ is a directed graph $G_k=(V_k,E_k)$, where the set of nodes $V_k=\Sigma ^{k-1}$ is the set of length-$(k-1)$ strings over $\Sigma$. There is an edge $(x,z)\in E_{k}$ if and only if the length-$(k-2)$ suffix of *x* is the length-$(k-2)$ prefix of *z*. There is therefore a natural correspondence between an edge (*x*, *z*) and the length-*k* string $x[1]x[2]\ldots x[k-1]z[k-1]$, where *x*[*i*] denotes the *i*th letter of *x* and similarly for *z*. Analogously, forbidden edges correspond to forbidden patterns of length *k*. We will thus sometimes abuse notation and write $S_k\subset E_k$. In this setting, one MVRS instance reduces to constructing a shortest path from the length-$(k-1)$ suffix of *u* to the length-$(k-1)$ prefix of *v* (the left- and right-context of the missing value) without using edges $S_k\subset E_k$ (the forbidden patterns).

### Example 1

**Fig. 1:**
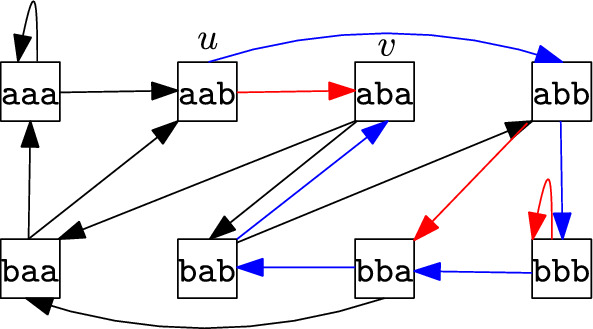
The complete de Bruijn graph $G_k=(V_k,E_k)$ of order $k=4$ over $\Sigma =\{\texttt {a},\texttt {b}\}$; forbidden edges are in red

(MVRS problem with length-*k* forbidden patterns) Consider a string aab#aba, where $\#$ denotes a missing value, and the following instance of the MVRS problem: $k=4$, $\Sigma =\{\texttt {a},\texttt {b}\}$, $u=\texttt {aab}$ (left context), $v=\texttt {aba}$ (right context), and $S_k=\{\texttt {bbbb},\texttt {abba},\texttt {aaba}\}$ (set of forbidden patterns). Figure 1 shows the complete de Bruijn graph of order 4 over $\Sigma =\{\texttt {a},\texttt {b}\}$. There is one node for every string in $\Sigma ^3$. There is, for instance, an edge from $\texttt {bbb}$ to $\texttt {bba}$, since $\texttt {bb}$ is both a suffix of $\texttt {bbb}$ and a prefix of $\texttt {bba}$. The edge $\texttt {aab}$ to $\texttt {aba}$ corresponds to the forbidden pattern $\texttt {aaba}$. The MVRS instance is: Find a shortest path from $u=\texttt {aab}$ to $v=\texttt {aba}$ without using any of the forbidden edges $S_k=\{\texttt {bbbb},\texttt {abba},\texttt {aaba}\}$. Such a path is formed by following the edges in blue in Fig. 1. This path corresponds to the shortest string $\texttt {aabbbaba}$ that starts with $\texttt {aab}$, ends with $\texttt {aba}$, and avoids the set of forbidden patterns. Thus, $x=\texttt {aabbbaba}$ replaces the missing value with $\texttt {bb}$. This solution is indeed a shortest string that has $\texttt {aab}$ as a prefix, $\texttt {aba}$ as suffix, and no occurrence of a forbidden pattern.

In real-world string datasets, we may have a large number of missing values. This is, for instance, the case in string sanitization, which is the main application of MVRS we consider here. However, we emphasize that MVRS is a general combinatorial optimization problem, which is directly applicable to any domain beyond sanitization where a string contains missing values that must be replaced. String sanitization is motivated by the need to disseminate strings in a way that does not expose sensitive patterns. For instance, location-based service providers, insurance companies, and retailers outsource their data to third parties who perform mining tasks, such as similarity evaluation between strings, frequent pattern mining, and clustering (Hong et al 2012; Liu et al 2015; Gwadera et al 2013; Ajunwa et al 2016). Yet, this dissemination raises privacy concerns, stemming from the fact that sensitive patterns may be exposed, if they occur in the disseminated string. Examples of sensitive patterns are certain parts of DNA associated with diseases (Steinlein 2001), visits to places that reveal health conditions (Bonomi et al 2016), or phrases that reveal sensitive facts about trial participants in a legal document (Allard et al 2020). In this context, we use the terms *sensitive* or *forbidden* patterns interchangeably.

**Contributions**  We assume throughout an integer alphabet $\Sigma =[1,|\Sigma |]$, whose size $|\Sigma |$ is polynomial in the input size *n*. More formally, we assume $|\Sigma |=n^{\mathcal {O}(1)}$. We also assume the standard word RAM model of computations with a machine word size $w=\Theta (\log n)$ (Cormen et al 2009). We summarize our main contributions below.

**1.** We design an efficient algorithm to solve the MVRS problem, and prove that the algorithm works in $\mathcal {O}(|u|+|v|+||S||\cdot |\Sigma |)$ time and space.

Note that, as justified in Sect. 3, we can always assume that $|\Sigma |\le |u|+|v|+||S||+1$. In particular, if $\Sigma$ is of constant size, our algorithm solves the MVRS problem in *linear time*. Our algorithm also implies that when we have $|S_k|$ forbidden patterns of fixed length *k*, we solve the MVRS problem in $\mathcal {O}(|u|+|v|+k|S_k|\cdot |\Sigma |)$ time and space *without* explicitly constructing the complete de Bruijn graph, which would require $\Omega (|\Sigma |^k)$ time and space, as it has $\Theta (|\Sigma |^{k-1})$ nodes and $\Theta (|\Sigma |^k)$ edges. To arrive at our algorithm, we employ techniques from formal languages (e.g., deterministic finite automata) and combinatorial pattern matching (e.g., suffix trees). See Sect. 3.

**2.** We show how our algorithm for MVRS can be effortlessly applied to fully sanitize a private string *w*. Specifically, we introduce the Shortest Fully-Sanitized String (SFSS) problem. On a high level, in the SFSS problem, we are given a private string *w* and a set $S_{k}$ of length-*k* forbidden patterns, and we are asked to construct a string *y* which contains no occurrence of a forbidden pattern *and* is as close as possible to *w* with respect to the *k*-gram distance (Ukkonen 1992). We solve SFSS by reducing it to $d\le |w|$ special instances of MVRS. Our algorithm runs in $\mathcal {O}(|w| + d\cdot k|S_{k}|\cdot |\Sigma |)$ time using $\mathcal {O}(|w| + k|S_{k}|\cdot |\Sigma |)$ space. See Sect. 4.

**3.** We propose a methodology for sanitizing and clustering a collection of private strings. Clustering is one of the main tasks for publishing privacy-protected data (Fung et al 2009; Jha et al 2005) and sanitization is necessary to prevent the inference of sensitive patterns from such data. For example, clustering a collection of DNA sequences has numerous applications in molecular biology, such as “grouping transposable elements, open reading frames, and expressed sequence tags, complementing phylogenetic analysis, and identifying *non-reference representative sequences* needed for constructing a pangenome” (Girgis 2022). Similarly, in text analytics, clustering can group similar documents or sentences, each modeled as a string, to improve retrieval and support browsing (Aggarwal and Zhai 2012), while clustering a collection of location profiles, each modeled as a string, can help discover user intention and user interests (Li et al 2008). As mentioned above, in these domains, there are naturally strings that are sensitive (Steinlein 2001; Bonomi et al 2016; Allard et al 2020) and many methods for sanitizing such strings (Bernardini et al 2019, 2020a, b, 2021a; Mieno et al 2021) add a letter $\#$, which is not a member of the original alphabet $\Sigma$, to the input string. Then, each $\#$ must be replaced with letters from $\Sigma$ for privacy reasons. Motivated by this, we study how we can first meaningfully replace these $\#$’s and then how to effectively cluster the resultant strings. Our methodology utilizes our algorithm for SFSS to sanitize the strings in the collection; as a baseline, we also propose a greedy algorithm. Next, our methodology computes distances between pairs of sanitized strings using a new effective and efficiently computable measure we propose, which is based on the notion of *longest increasing subsequence* (Schensted 1961). Last, the computed distances are provided as input to a well-known clustering algorithm (Kaufman and Rousseeuw 1990; Schubert and Rousseeuw 2021a). See Sect. 5.

**4.** We perform an extensive experimental study using several real and synthetic datasets to demonstrate the effectiveness and efficiency of our methodology for sanitizing and clustering a collection of private strings. Our results show that our algorithm for SFSS performs sanitization with little or no impact on clustering quality. They also show that it performs: (1) equally well or even better than the state-of-the-art method (Bernardini et al 2021a), which however is only applicable to very short strings due to its quadratic time complexity in the input string length; and (2) significantly better in terms of effectiveness compared to our greedy baseline, which is however significantly faster. In addition, we show that existing missing value replacement methods are not appropriate alternatives to our SFSS algorithm because they construct strings with a large number of forbidden patterns. See Sect. 7.

We organize the rest of the paper as follows: we present the necessary preliminaries in Sect. 2; we discuss related work in Sect. 6; and we conclude the paper in Sect. 8. A preliminary version of the paper without Contributions 3 and 4 was published by a subset of the authors as Bernardini et al (2021b).

## Preliminaries

***Strings*** We start with some definitions and notation from Crochemore et al (2007). An *alphabet* $\Sigma$ is a finite nonempty set of elements called *letters*. We assume throughout an integer alphabet $\Sigma =[1,|\Sigma |]$, whose size $|\Sigma |$ is polynomial in the input size. A *string* $x=x[1]\ldots x[n]$ is a sequence of *length* $|x|=n$ of letters from $\Sigma$. The *empty* string, denoted by $\varepsilon$, is the string of length 0. The fragment $x[i\mathinner {.\,.}j]$ is an *occurrence* of the underlying *substring* $s=x[i]\ldots x[j]$; *s* is a *proper* substring of *x* if $x\ne s$. We also write that *s* occurs at *position* *i* in *x* when $s=x[i] \ldots x[j]$. A *prefix* of *x* is a fragment of *x* of the form $x[1\mathinner {.\,.}j]$ and a *suffix* of *x* is a fragment of *x* of the form $x[i\mathinner {.\,.}n]$. An *infix* of *x* is a fragment of *x* that is neither a prefix nor a suffix. The set of all strings over $\Sigma$ (including $\varepsilon$) is denoted by $\Sigma ^*$. The set of all length-*k* strings over $\Sigma$ is denoted by $\Sigma ^k$.

***String Indexes*** Let *M* be a finite nonempty set of strings over $\Sigma$ of total length *m*. The *trie* of *M*, denoted by $\textsf {TR}(M)$, is a deterministic finite automaton (DFA) that recognizes *M* and has the following features (Crochemore et al 2007). Its set of states (nodes) is the set of prefixes of the elements of *M*; the initial state (root node) is $\varepsilon$; the set of terminal states (leaf nodes) is *M*; and edges are of the form $(u,\alpha ,u\alpha )$, where *u* and $u\alpha$ are nodes and $\alpha \in \Sigma$. The size of $\textsf {TR}(M)$ is thus $\mathcal {O}(m)$. The *compacted trie* of *M*, denoted by $\textsf {CT}(M)$, contains the root node, the branching nodes, and the leaf nodes of $\textsf {TR}(M)$. The term compacted refers to the fact that $\textsf {CT}(M)$ reduces the number of nodes by replacing each maximal branchless path segment with a single edge, and it uses a fragment of a string $s\in M$ to represent the label of this edge in $\mathcal {O}(1)$ machine words. The size of $\textsf {CT}(M)$ is thus $\mathcal {O}(|M|)$. When *M* is the set of suffixes of a string *y*, then $\textsf {CT}(M)$ is called the *suffix tree* of *y*, and we denote it by $\textsf {ST}(y)$. The suffix tree of a string of length *n* over an alphabet $\Sigma =\{1,\ldots ,n^{\mathcal {O}(1)}\}$ can be constructed in $\mathcal {O}(n)$ time (Farach 1997). The *generalized suffix tree* of strings $y_1\ldots ,y_k$ over $\Sigma$, denoted by $\textsf {GST}(y_1,\ldots ,y_k)$, is the suffix tree of string $y_1\$_1\ldots y_k\$_k$, where $\$_1,\ldots ,\$_k$ are distinct letters not from $\Sigma$ (Ukkonen 1995).

***Randomized Algorithms*** We next recall some basic concepts on randomized algorithms (Motwani and Raghavan 1995). For an input of size *n* and an arbitrarily large constant *c* fixed prior to the execution of a randomized algorithm, the term *with high probability* (whp), or inverse-polynomial probability, means with probability at least $1-n^{-c}$. When we say that the time complexity of an algorithm holds with high probability, it means that the algorithm terminates in the claimed complexities with probability at least $1-n^{-c}$. Such an algorithm is referred to as *Las Vegas whp*. When we say that an algorithm returns a correct answer with high probability, it means that the algorithm returns a correct answer with probability at least $1-n^{-c}$. Such an algorithm is referred to as *Monte Carlo whp*.

## Missing value replacement in strings

In this section, we show how to solve the MVRS problem: given two strings $u,v\in \Sigma ^{*}$ and a set $S\subset \Sigma ^{*}$ of forbidden strings, construct a shortest string $x\in \Sigma ^{*}$ such that *u* is a prefix of *x*, *v* is a suffix of *x*, and no $s\in S$ occurs in *x*; or FAIL if no such *x* exists.

### Simple preprocessing

By $||S||=\sum _{s\in S}|s|$, we denote the total length of all strings in set *S*. We make the standard assumption that $\Sigma$ is a subset of $[1,|u| + |v| + ||S||+1]$. If this is not the case and $|\Sigma |$ is polynomially bounded, we can sort the letters (i.e., integers) appearing in *u*, *v*, or *S* using radix sort in $\mathcal {O}(|u| + |v| + ||S||)$ time and replace each letter with its rank; if the alphabet is larger than that, we use a static dictionary (hashtable) (Fredman et al 1984) to achieve this in $\mathcal {O}(|u| + |v| + ||S||)$ time whp. Note that all letters of $\Sigma$ which are neither in *u* nor *v* nor in one of the strings in *S* are interchangeable. They can therefore all be replaced by a single new letter, reducing the alphabet to a size of at most $|u| + |v| + ||S|| + 1$. We will henceforth assume that $\Sigma$ is such a *reduced alphabet*. Further note that the input size of the MVRS instance is $(||S|| + |u| + |v|)\log |\Sigma |$ bits or $||S|| + |u| + |v|$ machine words. We further assume that set *S* is *anti-factorial*, i.e., no $s_1\in S$ is a proper substring of another element $s_2\in S$. If that is not the case, we take the set without such $s_2$ elements to be *S*. This can be done in $\mathcal {O}(||S||)$ time by constructing the generalized suffix tree of the original *S* after reducing $\Sigma$ (Farach 1997). Finally, we will assume that no strings from *S* occur in *u* or *v*, as this would immediately imply that the problem has no feasible solution. This condition can be verified in $\mathcal {O}(|u|+|v|+\Vert S\Vert )$ time by constructing the generalized suffix tree of *u*, *v* and *S* (Farach 1997).

### Main idea

We say that a string *y* is *S*-*dangerous* if $y=\varepsilon$ or *y* is a proper prefix of an $s\in S$; we drop *S* from *S*-dangerous when this is clear from the context. Thus, a dangerous string can be a substring of *x*, since it is not an element of *S*. We aim to construct a labeled directed graph *G*(*D*, *E*), which represents all feasible solutions of MVRS , as follows. The set of nodes is the set *D* of dangerous strings. There exists a directed edge labeled with a letter $\alpha \in \Sigma$ in the set *E* of edges from node $w_1$ to node $w_2$, if the string $w_1\alpha$ is not in *S* and $w_2$ is the longest dangerous suffix of $w_1\alpha$. Thus, an edge tells us how we can extend a dangerous string so that the extended string is still not in *S*.

Recall that, in MVRS , *u* and *v* must be a prefix and a suffix of the output string *x*, respectively. We can divide two cases: either *u* and *v* have a nonempty suffix/prefix overlap *p* (i.e., $u=u'p$ and $v=pv'$) such that $u'pv'$ does not contain any occurrence of forbidden patterns, in which case $x=u'pv'$, or no such overlap exists.

For the non-overlap case, we set the longest dangerous string in *D* which is a suffix of *u* to be the *source* node. We do this to be able to successfully spell *u* in the graph, since *u* must be a prefix of *x*, and *x* must not contain any string from *S*. We set every node *w* such that *wv* does not contain a string of *S* to be a *sink* node. Thus, sink nodes are possible suffixes of *x*, since they all have *v* as a suffix and do not contain any string from *S*. A path from the source node to any sink node corresponds to a feasible solution of MVRS , since we can spell *u* through *G* to arrive at the source, by traversing the path edges we extend *u* without creating any forbidden strings, and when we arrive at a sink node *w*, we know that we can safely append *v* to it. A shortest path from the source node to any sink node corresponds to a shortest such *x*, in the cases where *u* and *v* are not allowed to overlap.

The overlap case is treated separately before the non-overlap case: we compute all suffix/prefix overlaps of *u* and *v* in $\mathcal {O}(|u|+|v|)$ time and return - if it exists - the string $u'pv'$ such that $u=u'p$, $v=pv'$, and *p* is the longest overlap such that $u'pv'$ does not contain any forbidden pattern. This condition can be enforced again by using *G*, as we describe later in this section. The algorithm has two main stages. In the first stage, we construct the graph *G*(*D*, *E*). In the second stage, we find the source and the sinks, and we construct a shortest string *x* by checking the two cases separately.

Crochemore et al (1998) showed how to construct a complete deterministic finite automaton (DFA) accepting strings over $\Sigma$, which do not contain any forbidden substring from *S*. This is precisely the directed graph *G*(*D*, *E*). 3 We show that this DFA has $\Theta (||S||)$ states and $\Theta (||S||\cdot |\Sigma |)$ edges in the worst case. Note that we could use this DFA directly to solve MVRS by multiplying it^4^ with the automaton accepting strings of the form *uwv*, with $w\in \Sigma ^*$: in this way, we would obtain another automaton accepting all strings of length at least $|u|+|v|$ starting with *u*, ending with *v* and not containing any element $s \in S$ as a substring. However, this product automaton would have $\mathcal {O}((|u|+|v|)||S||)$ nodes. We will instead show an efficient way to compute the appropriate source and sink nodes on *G*(*D*, *E*) in $\mathcal {O}(|u|+|v|+||S||)$ time, resulting in a total time of $\mathcal {O}(|u|+|v|+||S||\cdot |\Sigma |)$.

We start by showing how *G*(*D*, *E*) can be constructed efficiently for completeness.

### Constructing the graph

First, we construct the trie of the strings in *S* in $\mathcal {O}(||S||)$ time (Crochemore et al 2007). We merge the leaf nodes, which correspond to the strings in *S*, into one forbidden node $s'$. Note that all the other nodes correspond to dangerous strings. We can therefore identify the set of nodes with $D' = D \cup \{s'\}$. We turn this trie into an automaton by computing a transition function $\delta : D' \times \Sigma \rightarrow D'$, which sends each pair $(w, \alpha ) \in D \times \Sigma$ to the longest dangerous or forbidden suffix of $w\alpha$ and $(s', \alpha ) \in \{s'\} \times \Sigma$ to $s'$. We can then draw the edges corresponding to the transitions to obtain the graph *G*(*D*, *E*). To help constructing this transition function, we also define a failure function $f:D\rightarrow D$ that sends each dangerous string to its longest proper dangerous suffix, which is well-defined because the empty string $\varepsilon$ is always dangerous.

The trie already has the edges corresponding to $\delta (w,\alpha ) = w\alpha$ if $w\alpha \in D'$. We first add $\delta (s',\alpha ) = s'$, for all $\alpha \in \Sigma$. For the failure function, note that $f(\varepsilon ) = f(\alpha ) = \varepsilon$. To find the remaining values, we traverse the trie in a breadth-first manner. Let *w* be an internal node, that is, a dangerous string of length $\ell > 0$. Then

$$f(w) = \delta (f(w[1.{\mkern 1mu} .\ell - 1]),w[\ell ]){\text{ and }}\delta (w,\alpha ) = \left\{ {\begin{array}{*{20}l} {w\alpha } \hfill & {{\text{if }}w\alpha \in D^{\prime}} \hfill \\ {\delta (f(w),\alpha )} \hfill & {{\text{if }}w\alpha \notin D^{\prime}} \hfill \\ \end{array} } \right..$$

Note that this is well defined, because $w[1\mathinner {.\,.}\ell -1]$ and *f*(*w*) are dangerous strings shorter than *w*, so the corresponding function values are already known.

Once we have computed the transition function and created the corresponding automaton, we delete $s'$ and all its incident edges, thus obtaining *G*(*D*, *E*). To ensure that we can access the node $\delta (w, \alpha )$ in constant time, we implement the transition functions using arrays in $\Theta (|\Sigma |)$ space per array.

Observe that we need to traverse |*D*| nodes and compute $|\Sigma |+1$ function values at each node (one value for *f* and $|\Sigma |$ values for $\delta$). Every function value is computed in constant time, thus the total time for the construction step is $\mathcal {O}(||S||+|D|\cdot |\Sigma |)$.

#### Lemma 1

*G*(*D*, *E*) has $\Omega (||S||)$ states and $\Theta (||S||\cdot |\Sigma |)$ edges in the worst case. *G*(*D*, *E*) can be constructed in the worst-case optimal $\mathcal {O}(||S||\cdot |\Sigma |)$ time.

#### Proof

For the first part of the statement, consider the instance where *S* consists of all strings of the form *ww* with $w \in \Sigma ^{k}$. Then *S* contains $|S|=|\Sigma |^k$ strings of total length $\Vert S\Vert = 2k\cdot |\Sigma |^{k}$. Observe that no two strings from *S* have a common prefix longer than $k-1$, thus the trie of *S* has more than $(k+1)|\Sigma |^{k}$ nodes (e.g., if $\Sigma$ is binary the trie has exactly $2\cdot 2^k-1+k\cdot 2^k$ nodes). This implies that *G*(*D*, *E*) has more than $k|\Sigma |^{k}=\Theta (\Vert S\Vert )$ nodes, as we need to remove the $|\Sigma |^{k}$ leaves from the trie. Moreover, for this instance, all but $|\Sigma |^{k}$ nodes of *G* (the former parents of the leaves of the trie) have exactly $|\Sigma |$ outgoing edges, thus the total number of edges is $\Theta (\Vert S\Vert \cdot |\Sigma |)$. The second part of the statement follows from the construction above (see also (Crochemore et al 1998)). $\square$

### Constructing a shortest string

To find the source node, that is, the longest dangerous string that is a suffix of *u*, we start at the node of *G*(*D*, *E*) that used to be the root of the trie, which corresponds to $\varepsilon$, and follow the edges labeled with the letters of *u* one by one. This takes $\mathcal {O}(|u|)$ time. Finding the sink nodes directly would be more challenging. Instead, we can compute the *non-sink nodes*, i.e., those dangerous strings $d \in D$ such that the string *dv* has a forbidden string $s \in S$ as a substring.

To this end, we construct the generalized suffix tree of the strings in $S\cup \{v\}$. Recall from Sect. 2 that this is the compressed trie containing all suffixes of all strings in $S\cup \{v\}$. This takes $\mathcal {O}(||S||+|v|)$ time (Farach 1997): let us remark that this step has to be done only once. We then find, for each nonempty prefix *p* of *v*, all suffixes of all forbidden substrings that are equal to *p*. We do that by traversing the unique path from the leaf representing the whole *v* to the root of the suffix tree. There are no more than |*v*| nodes on this path, thus the whole process takes $\mathcal {O}(|v|+||S||)$ time (Farach 1997). For each such suffix *p*, we set the prefix *q* of the corresponding forbidden substring to be a non-sink node, i.e., we have that $qp\in S$, *q* is a non-sink, and *p* is a prefix of *v*. Recall that all proper prefixes of the elements of *S* are nodes of *G*(*D*, *E*), and so this is well defined. Any other node is set to be a sink node.

We divide two cases, depending on the solution length: $|x| \le |u| + |v|$ (Case 1) and $|x| > |u| + |v|$ (Case 2).

*Case 1:* $|x| \le |u| + |v|$. In this case, *u* and *v* have a suffix/prefix overlap: a nonempty suffix of *u* is a prefix of *v*. We can compute the lengths of all possible suffix/prefix overlaps in $\mathcal {O}(|u|+|v|)$ time and $\mathcal {O}(|u|+|v|)$ space by, for instance, first constructing the generalized suffix tree of *u* and *v* (Farach 1997) and then traversing the unique path from the leaf corresponding to the whole *v* to the root. We must still check whether the strings created by such suffix/prefix overlaps contain any forbidden substrings. We do that by starting at $\varepsilon$ in *G*(*D*, *E*) and following the edges corresponding to *u* one by one. For each followed edge, we check if we have reached a sink node. If we reach a sink after following *i* edges and we have that $u[i+1\mathinner {.\,.}|u|] = v[1\mathinner {.\,.}|u|-i]$, that is, $|u|-i$ is the length of a suffix/prefix overlap, then we output $x = u[1\mathinner {.\,.}i]v$ and halt. Proceeding in this way, the suffixes of *u* are processed in decreasing order of their length, thus longer suffix/prefix overlaps are considered before shorter ones. Spelling *u* in *G* and checking the above condition at the sinks requires $\mathcal {O}(|u|)$ total time.

*Case 2:* $|x| > |u| + |v|$. Suppose that Case 1 did not return any feasible path. We then use a breadth-first search on *G*(*D*, *E*) from the source node to the *nearest* sink node to find a path. If we are at a sink node after following a path spelling string *h*, then we output $x = uhv$ and halt. In the worst case, we traverse the whole *G*(*D*, *E*). It takes $\mathcal {O}(|E|) = \mathcal {O}(|D|\cdot |\Sigma |)$ time.

In case no feasible path is found in *G*(*D*, *E*), we report FAIL.

***Correctness*** By construction, paths in graph *G* ending at sink nodes correspond to all and only the strings over $\Sigma$ having *v* as a suffix and with no occurrences of forbidden substrings. In both Case 1 and Case 2, we only follow paths that start with *u* and end at a sink, thus we always return a feasible solution. Let us now show that the returned solution is always optimal. First, note that the algorithm correctly searches for solution strings *x* in Case 1 ($|x|\le |u|+|v|$) before processing Case 2 ($|x|> |u|+|v|$), which is only considered if no solution in Case 1 exists. Furthermore, in Case 1, the algorithm halts as soon as it finds a feasible solution: since longer suffix/prefix overlaps of *u* and *v* are considered before shorter ones, and the longer the overlap, the shorter the output string, if the algorithm returns *x* in Case 1, this is optimal. Similarly, in Case 2 the paths corresponding to feasible solutions are processed in order of increasing length, and the algorithm will output *x* corresponding to the shortest feasible path if it exists, or report FAIL otherwise. Since, in Case 2, longer paths correspond to longer solutions, the length of *x* is always minimized.

***Complexities*** Constructing *G*(*D*, *E*) takes $\mathcal {O}(||S||+|D|\cdot |\Sigma |)$ time (see also (Crochemore et al 1998)). Finding the source and all sink nodes takes $\mathcal {O}(|u|+|v|+||S||)$ time. Checking Case 1 takes $\mathcal {O}(|u| + |v|)$ time. Checking Case 2 takes $\mathcal {O}(|D| \cdot |\Sigma |)$ time. It should also be clear that the following bound on the size of the output holds: $|x|\le |u| + |v| + |D|$. The total time complexity of the algorithm is thus

$$\mathcal {O}(||S|| + |u|+|v| + |D|\cdot |\Sigma |)=\mathcal {O}(|u|+|v| + ||S||\cdot |\Sigma |).$$

#### Remark 1

By symmetry, we can obtain a time complexity of $\mathcal {O}(||S|| + |u|+|v| + |D_s|\cdot |\Sigma |)$, where $D_s$ is the set including $\varepsilon$ and the proper suffixes of forbidden substrings.

The algorithm uses $\mathcal {O}(||S||\cdot |\Sigma | + |u|+|v|)$ working space, which is the space occupied by *G*(*D*, *E*) and the suffix tree of *u* and *v*. We obtain Theorem 1:

#### Theorem 1

Given two strings $u, v \in \Sigma ^*$ and a finite set $S\subset \Sigma ^*$, MVRS can be solved in $\mathcal {O}(|u|+|v|+||S||\cdot |\Sigma |)$ time and space, where $||S||=\sum _{s\in S}|s|$.

### A full example

**Fig. 2:**
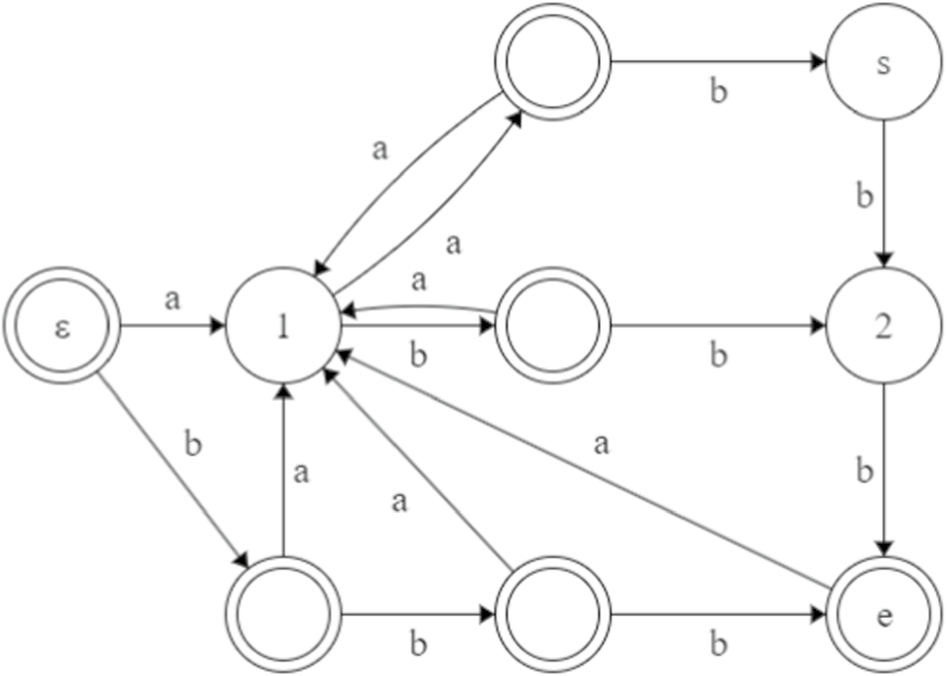
The graph *G*(*D*, *E*) after we have computed the source node marked *s* and the non-sink nodes marked *s*, 1, 2. All other nodes, marked with double circle, are sink nodes. The shortest path from source node to any sink node (the node marked *e*) is then $\texttt {bb}$, which gives the solution $x=\texttt {aab}\cdot \texttt {bb} \cdot \texttt {aba}$ ( $\cdot$ denotes concatenation)

In Fig. 2, we illustrate the automaton for the example in Fig. 1. Note that in this example, the automaton has more states than the corresponding complete de Bruijn graph, but for larger alphabets and larger values of *k* the opposite will be true. In particular, the complete de Bruijn graph has size $\Theta (|\Sigma |^k)$, while *G*(*D*, *E*) is always *guaranteed* to be of size $\mathcal {O}(\Vert S\Vert \cdot |\Sigma |)$ (Crochemore et al 1998), i.e., polynomial in the input size (recall that we have reduced to the case $\Sigma \subseteq [1,|u|+|v|+\Vert S\Vert +1]$).

Recall that $u=\texttt {aab}$, $v=\texttt {aba}$ and $S=\{\texttt {aaba,abba,bbbb}\}$. We start at node $\varepsilon$ of the automaton. After processing $u=\texttt {aab}$ we are at the source node (the node marked *s*). From the generalized suffix tree of *S* we find that the indexed suffixes $\texttt {aba}$ and $\texttt {a}$ are prefixes of $v=\texttt {aba}$. The complementary prefixes are $\texttt {a}$, $\texttt {aab}$ and $\texttt {abb}$, therefore the nodes marked *s*, 1, 2 are non-sink nodes, and all other nodes are sink nodes. Note that *u* and *v* have a suffix/prefix overlap (Case 1), and so we first check if the overlap string $\texttt {aaba}$ contains any string from *S*; indeed $\texttt {aaba}$ is itself a member of *S* and so *x* cannot be $\texttt {aaba}$. In fact, if from *s* we spell $\texttt {b}$, then we end up at node 2, a non-sink node. Hence we use breadth-first search (Case 2). The shortest path from *s* to any sink node (the node marked *e*) spells $\texttt {bb}$. Therefore $x=\texttt {aab}\cdot \texttt {bb} \cdot \texttt {aba}$ is a shortest string with prefix *u* and suffix *v* not containing any string from *S*.

#### Remark 2

The algorithm for obtaining Theorem 1 is fully deterministic when the original alphabet size is polynomial in the input size. As already noted at the beginning of Sect. 3, for larger alphabets, the preprocessing step to reduce the original integer alphabet to the integer interval $[1,\Vert S\Vert +|u|+|v|+1]$ uses a static dictionary (Fredman et al 1984), which requires a Las Vegas preprocessing step running in $\mathcal {O}(\Vert S\Vert +|u|+|v|)$ time whp.

In the following, we show that our result can be directly applied to solve a reachability problem in complete de Bruijn graphs in the presence of forbidden edges.

***Shortest Path in Complete de Bruijn Graphs*** Recall that the complete de Bruijn graph of order *k* over an alphabet $\Sigma$ is a directed graph $G_k=(V_k,E_k)$ with $V_k=\Sigma ^{k-1}$ and $E_{k}=\{(u,v)\in V_k\times V_k~|~u[1]\cdot v=u\cdot v[k-1]\}$. A *path* in $G_k$ is a finite sequence of elements from $E_k$, which joins a sequence of elements from $V_k$. By reachability, we refer to a path in $G_k$, which starts with a fixed *starting node* *u*, its infix is a sought (possibly empty) *middle path*, and it ends with a fixed *ending node* *v*. We consider this notion of reachability in $G_k$ in the presence of *forbidden edges* (or *failing edges*) represented by the set $S_k$ of forbidden length-*k* substrings over alphabet $\Sigma$.

We say that a subgraph $G_k^{S}=(V_k^{S},E_k^{S})$ of a complete de Bruijn graph $G_k$ *avoids* $S_k\subset \Sigma ^k$ if it consists of all nodes of $G_k$ and all edges of $G_k$ but the ones that correspond to the strings in $S_k$, that is, if $V_k^S=V_k$ and $E_k^{S}=E_k\setminus \{(u,v)\in E_k~|~u\cdot v[k-1]\in S_k\}$. Given $u,v\in \Sigma ^{k-1}$ and $S_k\subset \Sigma ^k$, it can be readily verified that there is a bijection between strings in $\Sigma ^n$ with prefix *u* and suffix *v* that do not contain any strings in $S_k$ and paths of length $n - k + 1$ that start at *u* and end at *v* in $G_k^{S}$.

**Table:** 

Shortest Path in de Bruijn Graphs Avoiding Forbidden Edges (SPFE)	
**Input:** The complete de Bruijn graph $G_k=(V_k,E_k)$ of order $k>1$ over an alphabet $\Sigma$, nodes $u,v\in V_k$, and a set $S_k\subset E_k$.	
**Output:** A shortest path from *u* to *v* avoiding any $e\in S_{k}$; or FAIL if no such path exists.	

Note that since $G_k$ is complete, it can be specified by *k* and $\Sigma$ in $\mathcal {O}(1)$ machine words. Theorem 1 yields the following corollary:

#### Corollary 1

Given the complete de Bruijn graph $G_k=(V_k,E_k)$ of order *k* over an alphabet $\Sigma$, nodes $u,v\in V_k$, and a set $S_k\subset E_k$, SPFE can be solved in $\mathcal {O}(k|S_{k}|\cdot |\Sigma |)$ time and space.

Note that Fig. 1 from Sect. 1 illustrates the same example as the one in Fig. 2, highlighting the difference between the de Bruijn graph perspective and the automaton perspective: explicitly constructing the complete de Bruijn graph would require $\Omega (|\Sigma |^k)$ time and space. (For compressed representations of de Bruijn graphs, see (Italiano et al 2021) and references therein.)

## Shortest fully-sanitized string

In Sect. 4.1, we motivate and formally define the Shortest Fully-Sanitized String (SFSS) problem. In Sect. 4.2, we present our algorithm for solving it.

### The problem

To support the dissemination of a string while preventing the exposure of a given set of sensitive patterns, a series of recent works (Bernardini et al 2019, 2020a, b, 2021a; Mieno et al 2021) investigated the problem of string sanitization: given a string *w* of length |*w*| over an alphabet $\Sigma$ and a set $S_k$ of $|S_k|$ length-*k* strings (patterns), construct a sanitized version *y* of *w* in which no pattern in $S_k$ occurs. We refer to patterns in $S_k$ as *forbidden* to emphasize how they are treated, and to $S_k$ as the *antidictionary* of the string. The aforementioned works consider an adversary who knows *y*, $\Sigma$, and $S_k$, and succeeds if, based on their knowledge, they can determine whether one or more patterns in $S_k$ occur in *w*. These works also impose some utility constraints and some objectives on *y*. Let $\mathcal {S}(w,S_k)$ denote the sequence of non-forbidden length-*k* substrings as they occur in *w* from left to right. To maintain the sequential structure of *w* as much as possible, all works imposed the constraint that $\mathcal {S}(w,S_k)$ is a subsequence of $\mathcal {S}(y,S_k)$.^5^ Depending on the targeted analysis task, they employed a different objective function, such as minimizing the edit distance of *w* and *y* or minimizing the *k*-gram distance of *w* and *y*.

The main common disadvantage of all existing works (Bernardini et al 2019, 2020a, b, 2021a; Mieno et al 2021) is that they do not *simultaneously* satisfy the following highly desirable requirements related to string sanitization:

*Req. 1*: String *w* should ultimately be *fully-sanitized*, i.e., the output string *y* must contain no occurrence of a forbidden pattern and no occurrence of any letter that is not in the original alphabet $\Sigma$. If only the former holds, *y* is called *partially-sanitized*.
*Req. 2*: The output string *y* should be as similar to *w* as possible (e.g., with respect to edit distance or some other similarity measure on strings, such as the *k*-gram distance).
*Req. 3*: The output string *y* should be constructed efficiently (e.g. in time linear or near-linear in the size |*w*| of the input string).

Requirement 1 is relevant to prevent the inference of forbidden patterns from *y* based on knowledge of the sanitization algorithm that produces *y* from *w* (Bernardini et al 2021a). Requirement 2 is relevant to preserve the sequential structure of *w*, which is important to accurately perform data analysis tasks that are based on sequence similarity. An example of such tasks is clustering, which aims to group a collection of strings into coherent groups (known as *clusters*) (Yang and Wang 2003). Requirement 3 is relevant to realize sanitization in practice since individual strings are typically long; e.g., an individual string could be a document written in natural language or a DNA sequence.

Specifically, none of the previous works (see Sect. 6) can fully sanitize a string *w* in polynomial time (i.e., satisfy Requirement 1), or efficiently construct a similar string to *w* under edit distance (i.e., satisfy Requirement 2 with edit distance *and* Requirement 3). These requirements have motivated us to formalize the task of constructing a *fully* sanitized string as the following combinatorial optimization problem.

**Table:** 

Shortest Fully-Sanitized String (SFSS)	
**Input:** A string $w\in \Sigma ^*$, an integer $k>1$, and a set $S_k\subset \Sigma ^{k}$.	
**Output:** A shortest string $y \in \Sigma ^*$ such that no $s\in S_k$ occurs in *y* and $\mathcal {S}(w,S_k)$ is a subsequence of $\mathcal {S}(y,S_k)$; or FAIL if no such *y* exists.	

We stress that SFSS is a general combinatorial optimization problem that applies to any domain involving a string which needs to be processed to satisfy Requirements 1, 2, and 3. Indeed, it is easy to see that these requirements are application-independent: for instance, in applications beyond sanitization (see Sect. 1), Requirement 1 should state that a string must not contain any occurrence of a forbidden pattern (e.g., domain-specific implausible patterns) or any missing value.

#### Example 2

Let ${\text{w = a}}\underline{{{\text{bbbb}}}} {\text{a}}\underline{{{\text{aaba}}}} {\text{a}}$, $\Sigma =\{{\texttt {a}},{\texttt {b}}\}$, $k=4$, and $S_k=\{{\texttt {bbbb}},{\texttt {aaba}},{\texttt {abba}}\}$. We have $\mathcal {S}(w,S_k)=\langle {\texttt {abbb},\texttt {bbba},\texttt {bbaa},\texttt {baaa},\texttt {aaab},\texttt {abaa}}\rangle$. All occurrences of forbidden patterns (strings from $S_k$) in *w* are underlined. A solution to the SFSS problem is string $y={\texttt {abbbaaabbbabaa}}$. Note that *y* is the shortest string in which no $s\in S_k$ occurs and $\mathcal {S}(w,S_k)$ is a subsequence of $\mathcal {S}(y,S_k)=\langle {\texttt {abbb},\texttt {bbba},\texttt {bbaa},\texttt {baaa},\texttt {aaab},\texttt {aabb},\texttt {abbb},\texttt {bbba},\texttt {bbab},\texttt {baba},\texttt {abaa}}\rangle$.

As mentioned above, in Sect. 4.2, we show an algorithm for solving SFSS in $\mathcal {O}(|w| + d\cdot k|S_{k}|\cdot |\Sigma |)$ time using $\mathcal {O}(|w| + k|S_{k}|\cdot |\Sigma |)$ space. Let us now briefly explain why the SFSS problem and our algorithm for solving it satisfy our three requirements.

Req. 1: String *y* is in $\Sigma ^*$. In Example 2, *y* is over $\Sigma =\{\texttt {a},\texttt {b}\}$, the original input alphabet.
Req. 2: We require that *y* is a shortest string that has $\mathcal {S}(w,S_k)$ as a subsequence of its *k*-gram sequence, as this implies that *y* has a long common subsequence of *k*-grams with *w*, and thus *w* and *y* are likely to be at small edit distance (Delcher et al 1999; Grossi et al 2016; Loukides and Pissis 2021). In Example 2, strings *w* and *y* share a subsequence $\mathcal {S}(w,S_k)$ of 6 4-grams and are at edit distance 4 (since $\texttt {a}$ in red replaces $\texttt {b}$ and three $\texttt {b}$’s in red are inserted): 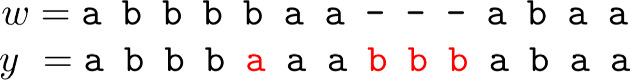 In fact, it is easy to prove that SFSS minimizes the *k*-gram distance between *w* and *y*, effectively making the length of the common subsequence of *k*-grams as long as possible relatively to the length of *y*. Recall that *y* is a string over the original alphabet $\Sigma$ containing no forbidden pattern as a substring.
Req. 3: Note that *k* is typically small (Bernardini et al 2021a, 2020a; Mieno et al 2021; Bernardini et al 2020b). Thus, if *d* and $\Sigma$ are reasonably small too, the algorithm is both time- and space-efficient.

### The algorithm

In this section, we show how to solve the $\text {SFSS}$ problem: given a string $w\in \Sigma ^n$, an integer $k>1$, and a set $S_k\subset \Sigma ^{k}$ of forbidden strings, construct a shortest string $y \in \Sigma ^*$ such that no $s\in S_k$ occurs in *y* and $\mathcal {S}(w,S_k)$ is a subsequence of $\mathcal {S}(y,S_k)$; or FAIL if no such *y* exists. We achieve this goal by solving multiple instances of the MVRS problem. Specifically, we show that Corollary 1 can be applied on the output of the TFS problem, introduced by Bernardini et al (2019), to solve the SFSS problem.

The TFS problem asks, given a string $w\in \Sigma^n$, an integer $k>1$, and a set of forbidden strings $S_k\subset \Sigma ^{k}$, to compute a shortest string $x \in \Sigma ^*$ such that no $s\in S_k$ occurs in *x* and $\mathcal {S}(w,S_k)=\mathcal {S}(x,S_k)$, or report FAIL if no such *x* exists. Bernardini et al (2019) showed that the solution to TFS is unique and is always of the form $x=x_0\#_1x_1\#_2\cdots \#_dx_d$, where $d\in [0,n]$, $\#_i$ denotes the *i*th occurrence of a symbol $\#\notin \Sigma$ for $i\in [1,d]$, with $x_i\in \Sigma ^*$ and $|x_i|\ge k$. It is easy to see why: if we had an occurrence of $\#_{i}x_i\#_{i+1}$ with $|x_i|\le k-1$ in *x* then we could have deleted $\#_{i}x_i$ to obtain a shorter string *x*, which is a contradiction. Furthermore, *d* is always upper bounded by the total number of occurrences of strings from $S_k$ in *w*, and it holds $|x|\le |w|+dk$ Bernardini et al (2019). Let us summarize the results related to string *x* from Bernardini et al (2019).

#### Theorem 2

(Bernardini et al (2019)) Let *x* be a solution to the TFS problem. Then *x* is unique, it is of the form $x=x_0\#_1x_1\#_2\cdots \#_dx_d$, with $x_i\in \Sigma ^*$, $|x_i|\ge k$, and $d\le n$, it can be constructed in the optimal $\mathcal {O}(n + k|S_k| + |x|)$ time, and $|x|=\Theta (nk)$ in the worst case.

Since $|x_i|\ge k$, each $\#$ replacement in *x* with a string from $\Sigma ^*$ can be treated *separately*. In particular, an instance $x_i\#_{i+1}x_{i+1}$ of this problem can be formulated as a shortest path problem in the complete de Bruijn graph of order *k* over alphabet $\Sigma$ in the presence of forbidden edges. Corollary 1 can thus be applied *d* times on $x=x_0\#_1x_1\#_2\cdots \#_dx_d$ to replace the *d* occurrences of $\#$ in *x* and obtain a final string over $\Sigma$: given an instance $x_i\#_{i+1}x_{i+1}$, we set *u* to be the length-$(k-1)$ suffix of $x_i$ and *v* to be the length-$(k-1)$ prefix of $x_{i+1}$. Let us denote by *y* the string obtained by this algorithm.

#### Example 3

Let $w={\texttt {a\underline{bbbb}a\underline{aaba}a}}$, $\Sigma =\{{\texttt {a}},{\texttt {b}}\}$, $k=4$, and $S_k=\{{\texttt {bbbb}},{\texttt {aaba}},{\texttt {abba}}\}$ (the instance from Example 2), and let x = abbbaaab#abaa be the solution of the TFS problem. By setting $u=\texttt {aab}$ and $v=\texttt {aba}$ in the SPFE problem, we obtain as output the path corresponding to string $p=\texttt {aabbbaba}$. The prefix $\texttt {aab}$ of *p* corresponds to the starting node *u*, its infix $\texttt {bb}$ corresponds to the middle path found, and its suffix $\texttt {aba}$ corresponds to the ending node *v*. We use *p* to replace $\texttt {aab\#aba}$ and obtain the final string $y={\texttt {abbbaaabbbabaa}}$.

However, to prove that *y* is a solution to the SFSS problem, we further need to prove that $\mathcal {S}(w,S_k)$ is a subsequence of $\mathcal {S}(y,S_k)$, and that *y* is a shortest possible such string.

#### Lemma 2

Let $x=x_0\#_1x_1\#_2\cdots \#_dx_d$, with $x_i\in \Sigma ^*$ and $|x_i|\ge k$, be a solution to the TFS problem on a string *w*, and let *y* be the string obtained by replacing the occurrences of $\#_1, \ldots , \#_d$ with the algorithm underlying Corollary 1. String *y* is a shortest string over $\Sigma$ such that $\mathcal {S}(w,S_k)$ is a subsequence of $\mathcal {S}(y,S_k)$ and no $s\in S_k$ occurs in *y*.

#### Proof

No $s\in S_k$ occurs in *y* by construction. With a slight abuse of notation, let $\mathcal {S}(x,S_k)$ be the sequence of *k*-grams over $\Sigma$ occurring in *x* from left to right. Since *x* is a solution to the TFS problem, we have that $\mathcal {S}(x,S_k)=\mathcal {S}(w,S_k)$. To show that $\mathcal {S}(x,S_k)=\mathcal {S}(w,S_k)$ is a subsequence of $\mathcal {S}(y,S_k)$, we must guarantee that (i) no *k*-gram is lost in the solution of the MVRS instances, and (ii) their order is preserved. For (i), note that some occurrences of *k*-grams from strings *u* and *v* input to MVRS do not appear in the output *x* only when $|x|\le |u|+|v|-k$, i.e., Case 1 of the algorithm is applied with an overlap longer than $k-1$: for shorter overlaps, the sequence of *k*-grams of *x* is a supersequence of those of $u\cdot v$. Since all of the *d* instances of MVRS have both *u* and *v* of length $k-1$, no overlap longer than $k-1$ exists, thus (i) holds; (ii) follows directly from $x_0,x_1,\ldots ,x_d$ occuring in *y* in the same order as in *x* by construction.

We now need to show that there does not exist another string $y'$ shorter than *y* such that $\mathcal {S}(w,S_k)$ is a subsequence of $\mathcal {S}(y',S_k)$ and no $s\in S_k$ occurs in $y'$. Suppose for a contradiction that such a shorter string $y'$ does exist. Since *x* is such that $\mathcal {S}(x,S_k)=\mathcal {S}(w,S_k)$ and no $s\in S_k$ occurs in *x*, $\mathcal {S}(x,S_k)$ forms also a subsequence of $\mathcal {S}(y',S_k)$ by hypothesis. Let $y[\ell _i\mathinner {.\,.}r_i]$ and $y'[\ell '_i\mathinner {.\,.}r'_i]$ be the shortest substrings of *y* and $y'$, respectively, where the *k*-grams of $x_i$ and $x_{i+1}$ appear and such that $|y[\ell _i\mathinner {.\,.}r_i]|>|y'[\ell '_i\mathinner {.\,.}r'_i]|$ (there must be an *i* such that this is the case, as we supposed $|y|>|y'|$). Since $y[\ell _i\mathinner {.\,.}r_i]$ is obtained by applying Corollary 1 to the length-$(k-1)$ suffix of $x_i$ and the length-$(k-1)$ prefix of $x_{i+1}$, it is a shortest string that has $x_i$ as a prefix and $x_{i+1}$ as a suffix, implying that $y'[\ell '_i\mathinner {.\,.}r'_i]$ can only be shorter if it is not of the same form: have $x_i$ as a prefix and $x_{i+1}$ as a suffix. Suppose then that $x_i$ is not a prefix of $y'[\ell '_i\mathinner {.\,.}r'_i]$, and thus there exist two *k*-grams of $x_i$ that are not consecutive in $y'[\ell '_i\mathinner {.\,.}r'_i]$. But then it is always possible to remove any letters between the two in $y'[\ell '_i\mathinner {.\,.}r'_i]$ to make them consecutive and obtain a string shorter than $y'[\ell '_i\mathinner {.\,.}r'_i]$. This operation does not introduce any occurrences of some $s\in S_k$, as the two *k*-grams are consecutive in $x_i$ which, in turn, does not contain any $s\in S_k$. By repeating this reasoning on any two *k*-grams of $x_i$ and $x_{i+1}$, we obtain a string $y_i''$ that has $x_i$ as a prefix, $x_{i+1}$ as a suffix and such that $|y_i''|<|y'[\ell '_i\mathinner {.\,.}r'_i]|<|y[\ell _i\mathinner {.\,.}r_i]|$. This is a contradiction, as $y[\ell _i\mathinner {.\,.}r_i]$ is a shortest string that has $x_i$ as a prefix and $x_{i+1}$ as a suffix. $\square$

By Theorem 2, Corollary 1, and Lemma 2, we have obtained Theorem 3:

#### Theorem 3

Let $d\le |w|$ be the total number of occurrences of strings from $S_k$ in *w*. The SFSS problem can be solved in $\mathcal {O}(|w| + d\cdot k|S_{k}|\cdot |\Sigma |)$ time using $\mathcal {O}(|w| + k|S_{k}|\cdot |\Sigma |)$ space.

#### Remark 3

The algorithm for obtaining Theorem 3 uses Corollary 1 (which relies on Theorem 1). It is thus deterministic if $|\Sigma |$ is polynomially bounded in the input size, otherwise, it is Las Vegas whp; see Remark 2.

We stress that the fact that *y* is the shortest possible is important for utility. The *k*-gram distance is a pseudometric that is widely used (especially in bioinformatics), because it can be computed in linear time in the sum of the lengths of the two strings (Ukkonen 1992). It is now straightforward to see that the *k*-gram distance between strings *w* (input of SFSS) and *y* (output of SFSS), such that $\mathcal {S}(w,S_k)$ is a subsequence of $\mathcal {S}(y,S_k)$ and no $s\in S_k$ occurs in *y*, is minimal. Thus, conceptually, SFSS introduces in *y* the least amount of spurious information, satisfying Requirement 2 of string sanitization (see Sect. 4.1).

#### Remark 4

By definition, SFSS uses a *single* antidictionary to replace all *d* occurrences of the letter $\#$. However, one can easily use a different antidictionary for every of the *d* occurrences of $\#$ without affecting the time complexity of our algorithm, since we “pay” for the whole antidictionary size in each of the *d* instances (see Theorem 3).

## Sanitizing and clustering private strings

Clustering a collection of sanitized strings is important to enable a range of applications in domains such as molecular biology, text analytics, and mobile computing (see Sect. 1). Meanwhile, the sanitized strings produced by many recent algorithms (Bernardini et al 2019, 2020a, b, 2021a; Mieno et al 2021) contain $\#$’s that must not appear in the clustered data, since they reveal the location of sensitive patterns. To address this issue and produce a high-quality clustering result, we employ our algorithm in Sect. 4, which solves the SFSS problem to ensure that $\#$’s are not present in the data, and develop a methodology for sanitizing a collection $\{w_1, \ldots , w_N\}$ of *N* strings in a way that preserves clustering quality. Clustering quality is captured by the well-known *K*-median problem (Kariv and Hakimi 1979; Ackermann et al 2010), as it will be explained in Sect. 5.3; informally, the clustering of $\{w_1, \ldots , w_N\}$ into *K* clusters, for some integer $K>0$, must be similar to the clustering of the strings $\{y_1, \ldots , y_N\}$ into *K* clusters, where $y_i$ is the sanitized version of $w_i$, for all $i\in [N]$. As an alternative to our algorithm for SFSS, we also present a baseline which performs full sanitization by *deleting* letters from the occurrences of the forbidden patterns – in this context, we call them *sensitive* patterns.

Our methodology is comprised of three phases: (I) We solve the SFSS problem with input each string $w_i$ in the collection of strings and the same *k* and set $S_k$ of forbidden patterns. This creates a collection of strings with no occurrence of $\#$. (II) We directly compute distances between each pair of the strings that are output in Phase I, by employing an effective and efficiently computable measure. (III) We give these distances as input to a well-known clustering algorithm.

In the following, we discuss each phase in detail.

### Phase I: Sanitization

We employ the algorithm for solving the SFSS problem (Sect. 4), which we denote by SFSS-ALGO. This encapsulates the algorithm underlying Theorem 1 for solving MVRS (Sect. 3). We apply SFSS-ALGO to each string of the input collection of strings separately, using the same *k* and set $S_k$ of forbidden patterns.

We also design, as an alternative, a baseline algorithm, referred to as GFSS (for Greedy Fully-Sanitized String). The main idea of GFSS is to read *w* in a streaming fashion and sanitize a forbidden pattern as soon as it arrives, by deleting the last letter (i.e., the one making it forbidden). It should be clear that the empty string $y=\varepsilon$ is a fully-sanitized string. As can be seen in Algorithm 1, GFSS appends letters from *w* to *y* from left to right as long as this does not introduce a forbidden pattern.

**Figure:**
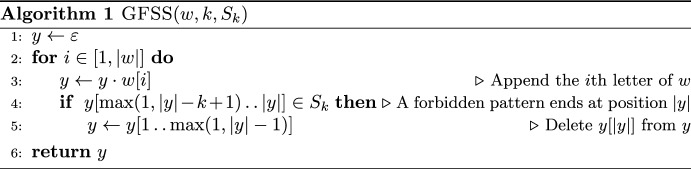
$$\text {GFSS} (w,k,S_k)$$

Algorithm 1 is correct in always producing a fully-sanitized string because, for any string $x=x[1\mathinner {.\,.}|x|]$, if $x[1\mathinner {.\,.}|x|-1]$ is fully-sanitized and $x[|x|-k+1\mathinner {.\,.}|x|] \notin S_k$, then *x* is also fully-sanitized. However, GFSS is not guaranteed to construct a feasible solution to the SFSS problem, since $\mathcal {S}(w,S_k)$ may not be a subsequence of $\mathcal {S}(y,S_k)$. For instance, in Example 2, GFSS produces $y=\texttt {abbb-aaab{-}-}$ (for ease of reference the deleted letters have been replaced with $\texttt {-}$). It is easy to see that $\mathcal {S}(w, S_k)$ in Example 2 is not a subsequence of $\mathcal {S}(y,S_k)=\texttt {abbb},\texttt {bbba},\texttt {bbaa},\texttt {baaa},\texttt {aaab}$.

GFSS is a good baseline because it removes an occurrence of a forbidden pattern by deleting at most one letter from *w*. In particular, when such occurrences are sparse in *w*, GFSS is often optimal in minimizing the edit distance of *y* to *w*. Moreover, GFSS is extremely fast in practice as we explain next. GFSS can be implemented in linear $\mathcal {O}(|w|+k|S_k|)$ time (Monte Carlo whp) by using Karp-Rabin fingerprints (KRFs) (Karp and Rabin 1987), a rolling hashing method that associates integers to strings in such a way that, with high probability, no collision occurs among the (length-*k*) substrings of a given string. The KRFs for all the length-*k* substrings of a string *w* can be computed in $\mathcal {O}(|w|)$ total time (Karp and Rabin 1987), and the KRFs of the strings from $S_k$ can be computed in $\mathcal {O}(k|S_k|)$ total time. To achieve the claimed time complexity, GFSS stores the KRFs of the strings from $S_k$ in a static dictionary (hashtable); it then reads the length-*k* substrings of *w* from left to right. For each such substring *s*, GFSS computes its KRF, searches it in the hashtable, and only appends the last letter of *s* to the output string if the KRF is not found. Thus, GFSS is expected to be much faster than SFSS (and hence also much faster than the edit distance based methods in Table 1).

### Phase II: Distance matrix computation

Given a string *x* and an integer $k>0$, we denote the sequence of length-*k* substrings as they occur from left to right in *x* by $S_k(x)$. Given a string *y*, we denote the list of occurrences of $S_k(x)[i]$ in *y* by $Occ_y(S_k(x)[i])$, and their concatenation for all $i\in [1,|x|-k+1]$ in order by $Occ_y(S_k(x))$. These lists can be computed in time $\mathcal {O}(|x|+|y|)$ by constructing the generalized suffix tree of *y* and *x* (Farach 1997). By $\text {LIS} _k(x,y)$, we denote the length of a *longest increasing subsequence* (that is, a longest subsequence such that all elements of the subsequence are in strictly increasing order) of the sequence:

$$Occ_y(S_k(x))=Occ_y(S_k(x)[1])\cdot Occ_y(S_k(x)[2])\cdots Occ_y(S_k(x)[|x|-k+1]).$$

From there on, to compute $\text {LIS} _k$, we make use of the algorithm of Schensted (1961) that takes $\mathcal {O}(h\log h)$ time for any length-*h* sequence. The $\text {LIS} _k(x,y)$ notion is widely used for efficient and effective sequence comparison (especially in bioinformatics (Delcher et al 1999)), as it is a good proxy for the edit distance of *x* and *y*. This is because when *x* and *y* have a large $\text {LIS} _k(x,y)$ value they are likely to be at small edit distance. The computation of edit distance between *x* and *y* requires $\mathcal {O}(|x||y|)$ time using dynamic programming (Crochemore et al 2007); and, unfortunately, there is good evidence (Backurs and Indyk 2018) suggesting that the textbook algorithm cannot be significantly improved. We next provide an example for $\text {LIS} _k(x,y)$.

#### Example 4

Consider the strings $x = \texttt {abbbbaaabaa}$, $y = \texttt {abbbaaabbbabaa}$ and let $k = 4$. All substrings of length *k* in *x* and *y* can be listed as follows:

$$S_k(x)=[\texttt {abbb, bbbb, bbba, bbaa, baaa, aaab, aaba, abaa}]$$

$$S_k(y)=[\texttt {abbb, bbba, bbaa, baaa, aaab, aabb, abbb, bbba, bbab, baba, abaa}].$$

To compute $\text {LIS} _k(x,y)$, we search for each element in $S_k(x)$, and get its list of occurrences in $S_k(y)$. In this example, we get $Occ_y(S_k(x)[1]) = [1,7]$, $Occ_y(S_k(x)[3]) = [2,8]$, $Occ_y(S_k(x)[4]) = [3]$, $Occ_y(S_k(x)[5]) = [4]$, $Occ_y(S_k(x)[6])=[5]$, $Occ_y(S_k(x)[8]) =[11]$, and $Occ_y(S_k(x)[2])=Occ_y(S_k(x)[7])=[]$. We construct the combined sequence:

$$\begin{aligned} Occ_y(S_k(x))&=Occ_y(S_k(x)[1]), Occ_y(S_k(x)[2]),\ldots , Occ_y(S_k(x)[8])\\&=\texttt {1,7,2,8,3,4,5,11}. \end{aligned}$$

The longest increasing subsequence in $Occ_y(S_k(x))$ is $\texttt {1,2,3,4,5,11}$, so $\text {LIS} _k(x,y) = 6$.

For $\text {LIS} _k(y,x)$, the nonempty occurrence lists are $Occ_x(S_k(y)[1])=[1]$, $Occ_x(S_k(y)[2])=[3]$, $Occ_x(S_k(y)[3])=[4]$, $Occ_x(S_k(y)[4])=[5]$, $Occ_x(S_k(y)[5])=[6]$, $Occ_x(S_k(y)[7])=[1]$, $Occ_x(S_k(y)[8])=[3]$, and $Occ_x(S_k(y)[11])=[8]$, so $Occ_x(S_k(y))=\texttt {1,3,4,5,6,1,3,8}$. The longest increasing subsequence in $Occ_x(S_k(y))$ is $\texttt {1,3,4,5,6,8}$, so $\text {LIS} _k(y,x) = 6$.

Note that generally, $\text {LIS} _k(x,y) \ne \text {LIS} _k(y,x)$. A simple example is when $x=\texttt {ab}$, $y=\texttt {ababababab}$, and $k=2$, where $\text {LIS} _2(x,y) = 5$ and $\text {LIS} _2(y,x) = 1$.

We next define a sequence comparison measure based on $\text {LIS} _k$ and use it as our distance measure. For two strings *x* and *y* and an integer $k>0$, we define this function as follows:

$$\mathcal {L}_k(x,y)=|x|+|y|-2(k-1) - \text {LIS} _k(x,y) - \text {LIS} _k(y,x).$$

In the following, we examine some properties of $\mathcal {L}_k$.

We first remark that $\mathcal {L}_k(x,y)$ does not satisfy the triangle inequality. For instance, consider the strings $x= \texttt {aaabaaab}$, $y=\texttt {abaaaaaa}$, $z = \texttt {aaaaaaaa}$, and $k=4$. Then $\mathcal {L}_4(x,y) = 6$, $\mathcal {L}_4(x,z) = 10$, $\mathcal {L}_4(y,z)=2$, whence $\mathcal {L}_4(x,z) > \mathcal {L}_4(x,y) + \mathcal {L}_4(y,z)$. Thus, the triangle inequality is not satisfied, and $\mathcal {L}_k$ is not a metric. The fact that $\mathcal {L}_k$ is not a metric implies that we cannot incorporate it in clustering algorithms whose objective function must be a metric (see (Ackermann et al 2010) for such algorithms for the *K*-median problem).

We next prove that $\mathcal {L}_k$ enjoys all properties of a pseudometric except for the triangle inequality.

#### Theorem 4

$\mathcal {L}_k(x,y)$ satisfies the following properties, for any strings *x*, *y* and any integer $0 < k \le \min (|x|,|y|)$:

1. $$\mathcal {L}_k(x,y)\ge 0$$;
2. $$\mathcal {L}_k(x,x)=0$$;
3. $$\mathcal {L}_k(x,y)=\mathcal {L}_k(y,x)$$.

#### Proof

We show each property separately.

1. By the definition of $\text {LIS} _k$, we have $\text {LIS} _k(x,x) = |x|-(k-1)$, which is the length of the sequence of all length-*k* substrings of *x*. This is because the longest increasing subsequence of $Occ_x(S_k(x))$ is clearly $1,\ldots ,|x|-k+1$, as $[1,|x|-k+1]$ is the range of positions of *x* where any length-*k* substring can occur. For any string *y* different from *x*, we cannot have a longest increasing subsequence of $Occ_x(S_k(y))$ longer than $|x|-(k-1)$, thus $\text {LIS} _k(y,x) \le \text {LIS} _k(x,x) = |x|-(k-1)$. We can thus rewrite $\mathcal {L}_k(x,y)$ as
  $$\mathcal {L}_k(x,y) = |x|-(k-1) + |y|-(k-1) - \text {LIS} _k(x,y) - \text {LIS} _k(y,x)$$
  $$= \text {LIS} _k(x,x) + \text {LIS} _k(y,y) - \text {LIS} _k(x,y) - \text {LIS} _k(y,x).$$
  Since $\text {LIS} _k(x,x) \ge \text {LIS} _k(y,x)$ and $\text {LIS} _k(y,y) \ge \text {LIS} _k(x,y)$, it follows that $\mathcal {L}_k(x,y) \ge 0$.
2. Since $\mathcal {L}_k(x,y) = \text {LIS} _k(x,x) + \text {LIS} _k(y,y) - \text {LIS} _k(x,y) - \text {LIS} _k(y,x)$, we get that $\mathcal {L}_k(x,x)=0$.
3. Trivial by the definition of $\mathcal {L}_k(x,y)$.

$$\square$$

Note that $\mathcal {L}_k(x,y) = 0$ does not imply $x=y$. For instance, consider $x=\texttt {aaa}$, $y=\texttt {aaaaaaa}$, and $k=3$. Then, $\text {LIS} _3(x,y)=5$, $\text {LIS} _3(y,x)=1$, and $\mathcal {L}_3(x,y) = 0$. Thus, $\mathcal {L}_k(x,y)$ is not a semimetric.

**Fig. 3:**
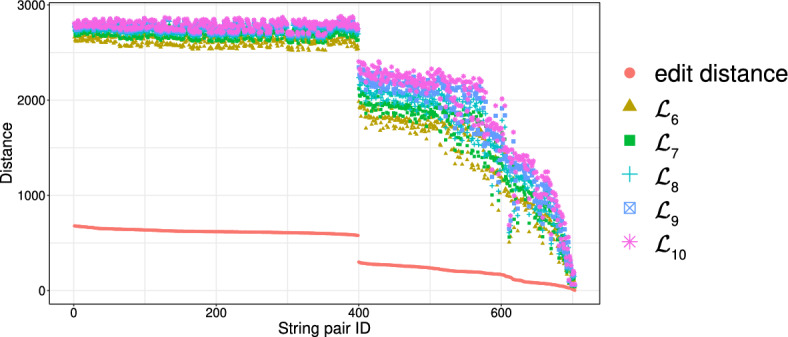
Edit distance and $\mathcal {L}_k$ distance, with $k\in [6,10]$, for each pair of strings in the Influenza dataset. The gap between both distance measures at string pair ID 400 is due to the underlying nature of the Influenza dataset. This dataset is comprised of five virus subtypes (H1N1, H2N2, H7N3, H7N9, H5N1). Sequence pairs within the same subtype or between closely related sutypes are highly similar (e.g., the pair with ID 618 comprised of two sequences of H2N2, or the pair with ID 419 comprised of one sequence in H1N1 and another in H5N1), whereas sequence pairs spanning other subtypes are not that similar (e.g., the pair with ID 26 comprised of one sequence in H1N1 and another in H2N2). Indeed, this is captured by both the edit distance and the $\mathcal {L}_k$ distance, $k\in [6,10]$, which have similar trends

It should thus be clear that a smaller value in $\mathcal {L}_k$ implies that *x* and *y* are more similar. To illustrate this, we have performed an experiment using the Influenza dataset (see Table 2a for its characteristics). In this experiment, we compared the distance of each pair of strings in the dataset, using first the edit distance and then $\mathcal {L}_k$ with each *k* in [6, 10]. We plot the results in Fig. 3: the *x* axis represents the string pairs in the dataset, in order of decreasing edit distance, and the *y* axis their distances; edit distance and $\mathcal {L}_k$ with each $k\in [6,10]$. Note that $\mathcal {L}_k$ tends to decrease when edit distance decreases, which indicates that a pair of similar strings with respect to edit distance will also be similar with respect to $\mathcal {L}_k$. To quantify the strength of the relationship between edit distance and $\mathcal {L}_k$, we applied the Kendall rank correlation coefficient test (Kendall 1938). The test uses the Kendall’s $\tau$ coefficient, which takes values in $[-1,1]$ and measures the relationship between two ranked variables. In our case, the first variable represents the edit distances of pairs of strings and the second $\mathcal {L}_k$ for the same pairs. A positive value for $\tau$ (respectively, $\tau =1$) signifies that the ranks of both variables increase (respectively, that the ranks of both variables are identical). The test results in the experiment of Fig. 3 for $k=6,7,8,9, 10$ are $\tau ={0.70, 0.67, 0.66, 0.65, 0.64}$ respectively, with *p*-value $p< 2.2\textrm{e}^{-16}$ (the null hypothesis is that edit distance and $\mathcal {L}_k$ for a given *k* are not related). The results thus indicate that edit distance and $\mathcal {L}_k$ distance have similar trends of change.

As a final step, we compute $\mathcal {L}_k(w_i,w_j)$ for every pair of strings $w_i, w_j$, such that $i\ne j$, in the input collection of strings, and fill up an $N\times N$ distance matrix with the values of $\mathcal {L}_k$.

### Phase III: Clustering

After sanitizing the sensitive patterns in Phase I and constructing the distance matrix in Phase II, we are ready to perform the actual clustering. We perform clustering following the well-known *K*-median clustering paradigm (Kariv and Hakimi 1979; Ackermann et al 2010). Intuitively, this paradigm seeks to find *K* representative strings in a given collection of strings, so as to minimize the sum of distances between each string in the collection and its closest representative string. That is, the objective of clustering is to minimize the error that is made by representing each string in the collection by its corresponding representative string. In our work, we quantify the distance between a pair of strings using our $\mathcal {L}_k$ measure.

This leads to the following clustering problem: Given an input collection of strings $\{y_1, \ldots , y_N\}$ and an integer $K>0$, find *K* strings $\{m_1, \ldots , m_K\}$ from the collection, so that $\sum _{i\in [N]}\min _{j\in [K]}\mathcal {L}_k(y_i, m_j)$ is minimized. The clusters are subsequently produced by assigning each string in the collection to its closest string from these *K* strings.

Since this problem is known to be NP-hard (Ackermann et al 2010), we employ the well-known Partitioning Around Medoids (PAM) (Kaufman and Rousseeuw 1990) heuristic. Specifically, we input the distance matrix constructed in Phase II to an efficient variant (Schubert and Rousseeuw 2021a) of PAM (Kaufman and Rousseeuw 1990), as it does not require the triangle inequality property (Kaufman and Rousseeuw 1990) and is effective in practice. Specifically, PAM can be used with any distance function (Schubert and Rousseeuw 2021b), i.e., a function *d* that is symmetric and for which $d(x,x)=0$ for all *x*, and thus it can be used with $\mathcal {L}_k$ (see Theorem 4).

PAM starts by an arbitrary selection of *K* strings in the input collection as the initial representatives (in PAM they are called medoids). It then selects randomly one representative and one non-representative string and swaps them if the cost of clustering $\sum _{c\in \mathcal {C}}\sum _{y\in c}\mathcal {L}{_k}(m_c,y)$ decreases, where *c* is a cluster in a clustering $\mathcal {C}$, $m_c$ is the representative of *c*, and *y* is a non-representative string in *c*. This is performed iteratively as long as the total cost of clustering decreases.

## Related work

The main application of our work we consider here is data sanitization (a.k.a. knowledge hiding), whose goal is to conceal confidential knowledge, so that it is not easily discovered by data mining algorithms.^6^ We thus review work on sanitizing strings and on sanitizing other data types in Sect. 6.1 and 6.2, respectively. The more fundamental problem we consider in this paper is missing value replacement in strings. We thus review work on missing value treatment in Sect. 6.3.

### String sanitization

There are several recently proposed approaches to sanitize a single string *w* (Bernardini et al 2019, 2020a, b, 2021a; Mieno et al 2021; Bernardini et al 2023); see Table 1. All these approaches are applied to a given set $S_k$ of length-*k* forbidden patterns and sanitize each such pattern by ensuring that it does not occur in the output string *y*. With the exception of (Bernardini et al 2020b), they perform partial sanitization. Specifically, they produce a string containing a letter, denoted by $\#$, that is not in the alphabet. Thus, it is not difficult for an adversary to locate the occurrences of forbidden patterns in the output string *y* and reverse the sanitization mechanism to produce *w* (Bernardini et al 2021a). On the other hand, the approach of (Bernardini et al 2020b) performs full sanitization.

**Table 1:** Existing string sanitization algorithms vs. our Theorem 3

**Algorithm**	**Sanitization**	**Minimization**	**Time complexity**
ETFS-RE (Bernardini et al 2021a)	Partial	Edit distance	$\mathcal {O}(|w|^2k|\Sigma |+k|S_k|)$
ETFS-DP (Bernardini et al 2020a)	Partial	Edit distance	$\mathcal {O}(|w|^2k+k|S_k|)$
ETFS-DP $^+$ (Mieno et al 2021)	Partial	Edit distance	$\mathcal {O}(|w|^2\log ^2k+k|S_k|)$
TFS (Bernardini et al 2021a)	Partial	*k*-gram distance	$\mathcal {O}(|w|+dk+k|S_k|)$
TFS+HM (Bernardini et al 2020b)	Full	ghost patterns	Polynomial s.t. conditions
Theorem 3 [This paper]	Full	*k*-gram distance	$\mathcal {O}(|w|+d\cdot k|S_k|\cdot |\Sigma |)$

Note that all algorithms satisfy the constraint that $\mathcal {S}(w,S_k)$ is a subsequence of $\mathcal {S}(y,S_k)$ so as to maintain the sequential structure of *w*; *d* denotes the number of occurrences of forbidden patterns in the input string *w*

The aforementioned approaches solve constrained optimization problems to preserve data utility, enforcing the following constraint: the output string *y* contains $\mathcal {S}(w,S_k)$, the sequence of all non-forbidden length-*k* substrings of *w*, as a subsequence of $\mathcal {S}(y,S_k)$. However, their optimization objectives differ.

Specifically, the ETFS-RE  (Bernardini et al 2021a), ETFS-DP  (Bernardini et al 2020a), and ETFS-DP $^+$  (Mieno et al 2021) algorithms optimize edit distance (subject to the constraint). The problem they solve is called ETFS (for Edit-distance, Total order, Frequency, Sanitization) and their name describes the main technique behind each of them. Specifically, ETFS-RE (RE is for Regular Expression) constructs a sanitized string by solving an approximate regular expression matching problem. That is, it first constructs a regular expression that encodes all ways in which the input string *w* can be sanitized and then constructs the sanitized string *y* that matches this regular expression with minimal edit distance from *w*. As can be seen in Table 1, the time complexity of ETFS-RE is quadratic in the length of *w* and linear in the size of the alphabet $\Sigma$. ETFS-DP (DP is for Dynamic Programming) removes the dependence from $|\Sigma |$ of the time complexity of ETFS-RE by avoiding actually constructing the regular expression. Instead, it exploits recurrences encoding the choices which specify the instance of the regular expression that is output. ETFS-DP $^+$ improves the time complexity of ETFS-DP, replacing $|w|^2k$ with $|w|^2\log ^2k$. This is done by advanced algorithmic techniques that reduce the redundancy and improve the efficiency of dynamic programming. Since *k* is a small constant in practice (Bernardini et al 2021a), the improvement is mostly of theoretical interest. Since ETFS-RE, ETFS-DP, and ETFS-DP $^+$ need time quadratic in the length of *w* and take space at least quadratic in the length of *w*, it is not practical to apply them to even moderately large strings (see Sect. 7). Even worse, as it was shown in Bernardini et al (2020a), there is no hope for a strongly subquadratic algorithm that solves ETFS, unless a famous computational hardness conjecture, called the Strong Exponential-Time Hypothesis (Impagliazzo et al 2001), is false.

The TFS algorithm (Bernardini et al 2021a) optimizes the *k*-gram distance (Ukkonen 1992) instead of the edit distance. It works by reading the string *w* from left to right and checking whether a length-*k* substring $s=w[i\mathinner {.\,.}i+k-1]$ is non-forbidden. If *s* is non-forbidden, then it is simply appended to *y*. Otherwise, TFS enforces two rules: (1) It appends the longest proper prefix of *s* (i.e., $w[i\mathinner {.\,.}i+k-2]$) followed by $\#$ and then by the longest proper suffix of *s* (i.e., $w[i+1\mathinner {.\,.}i+k-1]$); and (2) It removes $\#$ and the appended suffix, if this suffix is the same as the appended prefix. TFS is practical, as it requires time and space linear in the length of *w* and in the length of *y*. However, it only performs partial sanitization, as explained above. To address this issue, (Bernardini et al 2020b) recently proposed an Integer Linear Programming-based algorithm for replacing $\#$’s in the output of TFS. The algorithm, referred to as HM (for Hide and Mine), aims to preserve the utility in frequent length-*k* pattern mining. Specifically, it performs replacements that minimize the number of *ghost* patterns. 7 The time complexity of HM is polynomial only when certain conditions regarding the alphabet, *k*, and the number and position of these substrings hold; otherwise it is exponential in the size of the input.

Our work here differs from the aforementioned works along three important dimensions: (1) It applies full sanitization thereby better protecting *w*; (2) Its objective helps minimizing edit distance, which is computationally expensive to minimize directly; (3) It is efficient both in theory (i.e., it has a polynomial time complexity in all cases unlike HM) and in practice, as we show experimentally.

### Sanitizing other data types

There are numerous approaches to sanitize a collection of records. Each record can be a set of values (itemset) (Wu et al 2007; Gkoulalas-Divanis and Verykios 2009; Stavropoulos et al 2016; Lin et al 2018; Wu et al 2017), a sequence (Abul et al 2010; Gkoulalas-Divanis and Loukides 2011; Gwadera et al 2013; Bonomi et al 2016), a trajectory (Abul et al 2010), or a graph (Abul and Gökçe 2012). The forbidden patterns in these approaches are: itemsets in Wu et al (2007); Gkoulalas-Divanis and Verykios (2009); Stavropoulos et al (2016), subsequences in Abul et al (2010); Gkoulalas-Divanis and Loukides (2011); Gwadera et al (2013); Bonomi et al (2016), and graphs in Abul and Gökçe (2012). In addition, there is an approach for sanitizing a temporally annotated sequence (Loukides and Gwadera 2015), which aims to sanitize single letters. In terms of algorithmic techniques, these approaches employ integer programming (Gkoulalas-Divanis and Verykios 2009; Stavropoulos et al 2016), dynamic programming (Loukides and Gwadera 2015), or heuristics (Wu et al 2007; Abul et al 2010; Gkoulalas-Divanis and Loukides 2011; Gwadera et al 2013; Abul and Gökçe 2012; Bonomi et al 2016; Lin et al 2018; Wu et al 2017). Their goal is to: (1) reduce the frequency (support) of forbidden patterns, so that they cannot be mined from the output at a given frequency threshold; and (2) preserve data utility, often by preserving the set of frequent patterns that can be mined at a given frequency threshold (Gkoulalas-Divanis and Verykios 2009; Stavropoulos et al 2016; Wu et al 2007; Gkoulalas-Divanis and Loukides 2011; Gwadera et al 2013; Lin et al 2018; Wu et al 2017).

Our work here differs fundamentally from the aforementioned approaches: (1) in the type of forbidden patterns it considers (*substrings* vs. itemsets, subsequences, or graphs); and (2) in the type of utility it aims to preserve (*string similarity* vs. frequent pattern mining accuracy).

### Missing value treatment

A straightforward way to treat missing values is to delete them (Little and Rubin 2019; Enders 2010). However, deletion methods may incur excessive information loss (Enders 2010). An alternative to deletion is dealing directly with incomplete data (i.e., designing methods specifically for data that have missing values) (Calders et al 2007; Fiot et al 2007; Yu et al 2022; Figueroa et al 2008). Methods that directly deal with incomplete data are developed for problems other than string sanitization. For example, (Calders et al 2007; Fiot et al 2007) consider pattern mining and utilize interestingness measures suited to mining patterns with missing values. The work of Yu et al (2022) considers causal feature selection (Yu et al 2020) and proposes an approach for this problem. Last, the work of Figueroa et al (2008) considers clustering and proposes a clustering algorithm for a set of fixed-length binary strings. A third way to deal with missing values is missing value replacement (a.k.a. imputation) (Dong et al 2015; Tuikkala et al 2008; Bießmann et al 2018; Zhu et al 2021; Li et al 2015; Karmitsa et al 2022; Vreeken and Siebes 2008; Zhang et al 2019; Bansal et al 2021; Lin et al 2020; Ma et al 2020; Wellenzohn et al 2017; Bernardini et al 2023). Missing value replacement methods have the benefit that their output can be used in any task.

Missing value replacement methods have been proposed for different types of data, ranging from relational data with different types of attributes (Zhu et al 2021; Li et al 2015; Karmitsa et al 2022) to transaction data (Vreeken and Siebes 2008), time-series (Zhang et al 2019; Bansal et al 2021; Lin et al 2020), data streams (Ma et al 2020; Wellenzohn et al 2017), and strings (Li et al 2009; Li and Durbin 2010; Bernardini et al 2023). In the following, we discuss some of the most relevant missing value replacement methods for our setting. However, note that none of these methods outputs a string that solves our SFSS problem.

For DNA strings, it is common to replace a missing value with a fixed or randomly selected letter from the DNA alphabet $\{\texttt {A},\texttt {C},\texttt {T},\texttt {G}\}$. This strategy is employed by state-of-the-art DNA data processing tools  (Li et al 2009; Li and Durbin 2010). A more effective method working for any string has recently been proposed in Bernardini et al (2023). It can be seen as a generalization of the Hide and Mine (HM) method, discussed in Sect. 6.1. As in the HM method, the objective is to minimize the number of ghost patterns. The difference from the HM method is that two missing values do not need to be at least *k* positions apart in the input string. The method of Bernardini et al (2023) is based on Integer Linear Programming and is exponential in the number of missing values. Our work differs from Bernardini et al (2023) in: (1) the objective function; and (2) the fact that it has a polynomial time complexity.

A third approach (Halpin 2016, 2012, 2013) for missing value replacement in strings is called MIMR (for Multiple Imputation with Multinomial Regression). This approach is based on prediction and more specifically on a statistical technique, called *multiple imputation* (Rubin 1987). The main idea behind multiple imputation is to generate multiple plausible values, called *imputations*, for a variable (i.e., multiple letters, each of which can replace a missing letter in our setting) by making draws from the predicted distribution of the data multiple times and then using these plausible values to quantify the uncertainty of what the missing value might be. The benefit of using more than one imputation per value is increased accuracy in missing value replacement. The MIMR approach replaces the missing values iteratively: the candidate replacements for a missing value are the non-missing values that are immediately before and after it. Furthermore, the already replaced missing values at a certain iteration are treated as non-missing values and taken into account in the replacement of missing values in the next iterations. For example, if the input string to MIMR is ab###de, this approach will first replace the leftmost or rightmost missing value $\#$ with a letter and then use the replaced letter to decide how the remaining $\#$’s will be replaced. To predict the best imputation for a letter using the letters before and after it, MIMR uses a multinomial regression (Hilbe 2009) prediction model.

Another approach that is similar to MIMR in that it uses a prediction model is MissForest (Stekhoven and Bühlmann 2012). This approach differs from MIMR in that it uses a machine learning algorithm instead of a regression model. Specifically, MissForest trains a Random Forest (Breiman 2001) classifier on the non-missing values, which is used to predict the missing values. The classifier training and use is performed multiple times, until a convergence criterion regarding the quality of the output data is met. Another difference from MIMR is that MissForest makes no assumptions about the data distribution (Tang and Ishwaran 2017). A drawback of MissForest is that its basic implementation is not scalable to large datasets. Therefore, a faster implementation has been proposed in Tang and Ishwaran (2017).

Both MIMR and MissForest methods are well-established Fuller and Stecy-Hildebrandt (2015); McMunn et al (2015); Tang and Ishwaran (2017). Furthermore, MissForest has been shown (Stekhoven and Bühlmann 2012; Tang and Ishwaran 2017) to outperform many classic missing value replacement algorithms, such as KNNimpute (Troyanskaya et al 2001) and MICE (van Buuren and Groothuis-Oudshoorn 2011). MIMR, MissForest, KNNimpute, and MICE differ from our work in that they *do not explicitly consider forbidden patterns* and in that they *do not have the same quality requirements* as those in the SFSS problem. Consequently, they cannot guarantee that the strings they produce will not have forbidden patterns. In fact, as we show experimentally in Sect. 7, incorporating either MIMR or MissForest in methods for replacing missing values in our context is not satisfactory because it leads to strings containing a large number of forbidden patterns.

Last, we note that the main application of our work is to replace missing values ($\#$’s) in a string, in order to better preserve privacy. Missing value replacement has been extensively used for the same general purpose of preserving privacy on relational data (see e.g., (Rubin 1993; Raghunathan et al 2003)).

## Experimental evaluation

In this section, we evaluate our methodology (Sect. 5) in terms of effectiveness and efficiency:

- We measure *effectiveness* based on the similarity between clustering a collection of strings and the clustering produced after sanitizing the same collection of strings. In addition, we measure effectiveness based on how similar are the sanitized strings to their representative.
- We measure *efficiency* based on the runtime of a sanitization method.

In addition, we demonstrate that using well-established missing value replacement methods, namely MIMR and MissForest (see Sect. 6.3), as basis of heuristics for dealing with the SFSS problem is not appropriate. In particular, we show that it leads to strings with forbidden patterns.

### Datasets

We used both real and synthetic datasets, as described next.

***Real datasets*** We used five publicly available real datasets; each dataset is a collection of *N* strings, which we denote by $\{w_1, \ldots , w_N\}$. The characteristics of these datasets are shown in Table 2a. As can be seen, the datasets we used come from different domains and have quite different characteristics. The parameters used in experiments on these datasets are shown in Tables 2b and 2c. Note that these datasets were also used in many prior works on clustering (Nguyen et al 2018b; Steinegger and Söding 2018; Li et al 2017b; Kelil et al 2007; Anjum et al 2023).

We preprocessed the News and WebKb datasets to help their clustering by reducing their alphabet size to 100 8 and by removing an outlier 9 in WebKb.

In addition, we used substrings of two single strings to evaluate runtime. The first string is the chromosome 21 sequence of Homo Sapiens (Schneider et al 2016) and is referred to as Chr; the second string is a protein sequence (Suzek et al 2015) and is referred to as Prot. The length |*w*| of Chr is 46,709,983 letters and that of Prot is 16,000 letters. The alphabet size $|\Sigma |$ of Chr is 4 and that of Prot is 20.

***Synthetic datasets*** We used synthetic datasets to study the impact of different parameters. To generate each synthetic dataset, we first obtained *K* strings from a real dataset that have length 2000. These strings were used as seeds to initiate clusters, each comprised of *L* strings (including the seed string). To construct a cluster, we started from a seed string *Q* and generated $L-1$ strings $Q_1, \ldots , Q_{L-1}$ that we added to the cluster. Each of these strings is at edit distance at most *e* from *Q* since it was created by performing *e* edit distance operations on *Q*, each with equal probability. We used $K \in [5, 25]$ and $L\in [10,100]$. We varied *e* in [200, 1800] and report results for a normalized version of *e*, which is defined as $\delta = \frac{e}{|Q|}$, where $|Q|=2000$. We selected seed strings from two real datasets: (1) a DNA dataset obtained from Zhang and Zhang (2017); and (2) a protein dataset obtained from Suzek et al (2015).

**Table 2:** (a) Real datasets and their characteristics. Each dataset is a collection of strings. (b) and (c) Parameters and range of values used in these parameters (default values are in parenthesis)

Dataset	Domain	Alphabet size $|\Sigma |$	No. of strings *N*	Max string length	Mean string length
(a)					
News (Zhou et al 2016)	News	100*	4976	6779	140
WebKb (Nguyen et al 2018a)	Web	100*	4167	2082	133
Influenza (Li et al 2017b)	Virus	4	38	1467	1350
Moesm15 (Kelil et al 2007)	Proteins	20	316	2441	259
Hpc (log dataset 2022)	Log file	65	2000	368	44

(b)					
Dataset	No. of forbidden patterns $|S_k|$	No. of occurrences of forbidden patterns *d*	Pattern length *k*	No. of clusters *K*	
News	[80, 200]	[492, 1130]	[6, 14] ($\textbf{10}$)	5	
WebKb	[80, 200]	[470, 1013]	[6, 14] ($\textbf{10}$)	5	

(c)					
Dataset	Percentage of forbidden patterns $\mathcal {R}=\frac{|S_k|}{|\Sigma ^k|}$	Pattern length *k*	No. of clusters *K*		
Influenza	[5, 25] ($\textbf{10}$)	[6, 10] ($\textbf{8}$)	5		
Moesm15	[5, 25] ($\textbf{10}$)	[3, 5] ($\textbf{4}$)	28		
Hpc	[5, 25] ($\textbf{10}$)	[2, 4] ($\textbf{3}$)	8		

### Setup

SFSS-ALGO (or SFSS algorithm), which uses the output of TFS as input, constructs a fully sanitized string *y* that is at minimum *k*-gram distance from the original string *w* subject to the constraint: $\mathcal {S}(w,S_k)$ is a subsequence of $\mathcal {S}(y,S_k)$. We compared SFSS to ETFS-DP  (ETFS, in short) (Bernardini et al 2020a), which constructs a partially sanitized string *y* that is at minimum edit distance from the original string *w* subject to the same constraint: $\mathcal {S}(w,S_k)$ is a subsequence of $\mathcal {S}(y,S_k)$ (see Table 1). Since partial sanitization is insufficient to hide the locations of the confidential patterns (Bernardini et al 2019, 2021a), ETFS is not an alternative to our approach. However, we use it to evaluate the impact that using *k*-gram distance and full sanitization instead of edit distance and partial sanitization has on quality. Recall that ETFS-DP, ETFS-RE, and ETFS-DP $^+$ are exact algorithms, which construct a solution with the same optimal cost (minimum edit distance) in $\Omega (|w|^2)$ time. Yet, we chose ETFS-DP because it is significantly faster than ETFS-RE, and equally efficient in practice, but much easier to implement, than ETFS-DP $^+$. We did not compare against TFS+HM  (Bernardini et al 2020b), since it has a fundamentally different objective function (see Table 1). In addition, we compared against GFSS.

To capture the effectiveness of our methodology, we used the following measures: (1) Normalized Mutual Information (NMI); and (2) Adjusted Rand Index (ARI). NMI and ARI quantify the impact of sanitization on clustering by comparing a clustering *C*, which is comprised of |*C*| clusters and obtained from the original data, to another clustering $C'$, which is comprised of $|C'|$ clusters and obtained using the sanitized version of the same data. NMI and ARI are standard measures of clustering quality (Nguyen et al 2010; Meila 2007), which measure the similarity between *C* and $C'$. The values of NMI and ARI are in [0, 1] with larger values being preferred (more similar clusterings). A value of 1 in either measure implies that two clusterings *C* and $C'$ are identical.

Let *N* be the number of strings that *C* and also $C'$ are comprised of and $a_i$ (respectively, $b_i$) be the number of strings contained in the *i*th cluster of *C* (respectively, $C'$). NMI is defined as follows:

1 $$\begin{aligned} \text {NMI}(C,C')=\frac{I(C,C')}{\max (H(C),H(C'))}, \end{aligned}$$

where $H(C)=-\sum _{i=1}^{|C|}\frac{a_i}{N}\log (\frac{a_i}{N})$ (respectively, $H(C')$) is the entropy of *C* (respectively, $C'$), and $I(C,C')=\sum _{i=1}^{|C|}\sum _{j=1}^{|C'|}\frac{n_{ij}}{N}\log (N\frac{n_{ij}}{a_ib_j})$ is the joint entropy of *C* and $C'$, with $n_{ij}$ being the number of strings contained in both the *i*th cluster of *C* and the *j*th cluster of $C'$.

ARI is defined as follows:

2 $$\begin{aligned} ARI(C,C')=2\cdot \frac{N_{00}N_{11}-N_{01}N_{10}}{(N_{00}+N_{01})(N_{01}+N_{11})+(N_{00}+N_{10})(N_{10}+N_{11})}, \end{aligned}$$

where $N_{00}$ is the number of pairs of strings that are in different clusters in both *C* and $C'$, $N_{01}$ the number of pairs of strings that are in different clusters in *C* but in the same cluster in $C'$, $N_{10}$ the number of pairs of strings that are in the same cluster in *C* but in different clusters in $C'$, and $N_{11}$ the number of pairs that are in the same cluster in both *C* and $C'$. We also used the $\text {LIS} _k$ similarity measure (see Sect. 5.2) to evaluate how similar are the strings in each cluster of the sanitized dataset. Specifically, we report $\sum _{i\in [N]}\max _{j\in [K]}\left( \text {LIS} _k(y_i, m_j)+\text {LIS} _k(m_j, y_i)\right)$, where $y_i$ is a string in the sanitized dataset and $m_j$ is a cluster representative. We refer to this measure as $\text {LIS}$. Clearly, larger values in $\text {LIS}$ indicate a clustering of higher quality. We do not report results with the normalized version of $\text {LIS} _k(y_i, m_j)+\text {LIS} _k(m_j, y_i)$, denoted by $\mathcal {L}_k$ in Sect. 5.2, as the sanitized strings produced by the different algorithms we evaluate have different lengths, which makes the use of $\mathcal {L}_k$ inappropriate.

The clustering *C* was obtained by applying Phases II and III (see Sects. 5.2 and 5.3, respectively) to the input collection of the original strings. The clustering $C'$ was obtained by applying Phase I (i.e., applying a sanitization algorithm such as SFSS), followed by Phases II and III. Recall that Phases II and III use the *k*-gram based $\mathcal {L}_k$ measure. An approach that uses ETFS in Phase I and edit distance in Phases II and III is feasible but it would violate Req. 1 and Req. 3 in Sect. 4.1. That is, it would reveal the location of sensitive patterns and be inefficient. We do not consider it further, as it did not offer a benefit in terms of quality in our preliminary experiments.

Since the datasets do not come associated with forbidden patterns, we selected the forbidden patterns randomly, following previous works on data sanitization (Bernardini et al 2021a; Gkoulalas-Divanis and Loukides 2011; Gwadera et al 2013). In the cases of News and WebKb, the forbidden patterns had to occur in the dataset, in accordance with the setup of ETFS we compare against (Bernardini et al 2021a) (see Table 2b for details). In all other datasets, the forbidden patterns were selected from the set $\Sigma ^k$, the space of all possible length-*k* substrings that can be constructed from the alphabet $\Sigma$, to be able to consider a larger number of forbidden patterns (see Table 2c for details). We used 10 different sets of forbidden patterns.

We conducted all experiments on a server with an AMD Opteron^™^ Processor 6386 SE at 2.8 GHz and 252GB RAM. We used a single CPU of the server. Our source code is written in C++. The heuristics in Sect. 7.6 employ the standard R implementations of MIMR (Halpin 2016, 2012, 2013) and MissForest (Stekhoven and Bühlmann 2012); see Sect. 6.3 for a discussion of MIMR and MissForest. These implementations can be found in package (2022b) and package (2022a), respectively, and they were configured using their default values. The source code and all datasets we used can be found at https://github.com/YagaoLiu/SFSS. All experimental results have been averaged over 10 runs, and we report the mean of results. When the difference between two means (e.g., the mean NMI of ETFS vs the mean NMI of SFSS) is numerically small, we also report the obtained *p*-values from a *t*-test, used to determine if there is a significant difference between two means. A *p*-value smaller than 0.05 implies that the difference between the two means is statistically significant. The running time has been measured using the C++ class std::chrono::high_resolution_clock.

**Fig. 4:**
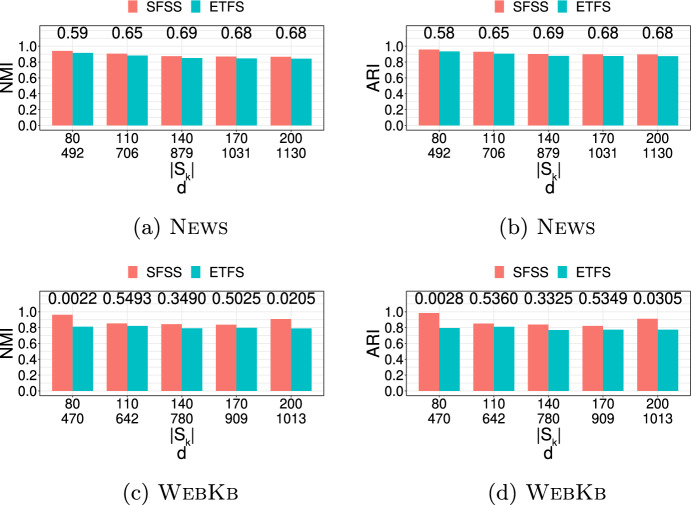
NMI and ARI for varying number $|S_k|$ of forbidden patterns, for:** a**,** b** News and** c**, ** d** WebKb. The NMI and ARI values, as well as $|S_k|$ and the number *d* of occurrences of forbidden patterns, are averages over 10 runs. On the top of each pair of bars, we plot the *p*-value of a *t*-test; $p< 0.05$ implies that the difference between ETFS and SFSS is statistically significant

**Fig. 5:**
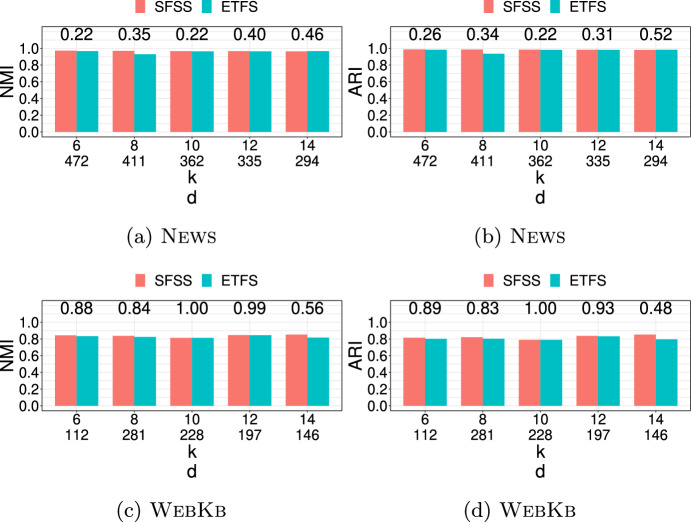
NMI and ARI for varying length *k* of forbidden patterns, for:** a**,** b** News and** c**,** d** WebKb. The NMI and ARI values, as well as the number *d* of occurrences of forbidden patterns, are averages over 10 runs. On the top of each pair of bars, we plot the *p*-value of a *t*-test; $p< 0.05$ implies that the difference between ETFS and SFSS is statistically significant

### Small antidictionary

In this section, we show that when the antidictionary is small, SFSS is able to preserve the clustering quality well, while being efficient. We compare it to ETFS whose effectiveness is not affected by the antidictionary size, since it does not replace the $\#$’s. The efficiency of SFSS and ETFS are affected in the same way by the antidictionary size. Note that for such a small antidictionary, GFSS performs perfectly because it is *trivial* for this algorithm to not reinstate any forbidden pattern (since there are very few patterns, deleting a letter removes an occurrence of a forbidden pattern without creating an occurrence of another forbidden pattern or of the forbidden pattern itself). Therefore, we have excluded GFSS from the experiments of this section. However, as we will show in the next section, GFSS performs much worse than SFSS (and ETFS) when the size of the antidictionary grows.

We demonstrate in Figs. 4 and 5 that both ETFS and SFSS are very effective at preserving clustering quality, as captured by both NMI and ARI. In particular, Fig. 4 shows the NMI and ARI scores for both methods, for varying number of forbidden patterns. Note that the NMI and ARI scores are very high. For example, the average NMI scores were 0.80 and 0.88 for ETFS and SFSS, respectively, and the corresponding ARI scores were 0.85 and 0.91, respectively. The differences between the two methods are not statistically significant, except in four cases (see the cases in Figs. 4a and 4b where the number on the top of bars is $<0.05$). In these cases, SFSS outperformed ETFS. Also, Fig. 5 shows the NMI and ARI scores for ETFS and SFSS, for varying length of forbidden patterns. Again, the two methods performed very well, and the differences between them are not statistically significant.

**Fig. 6:**
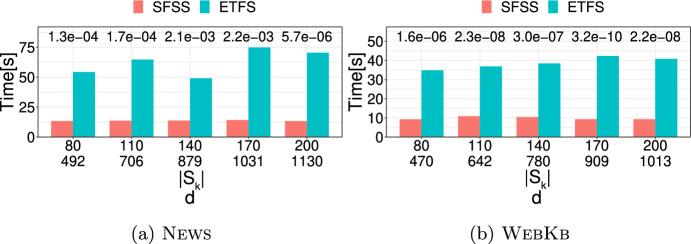
Runtime (secs) for varying number $|S_k|$ of forbidden patterns, for:** a** News and** b** WebKb. The runtime values, as well as the number *d* of occurrences of forbidden patterns, are averages over 10 runs. On the top of each pair of bars, we plot the *p*-value of a *t*-test; $p< 0.05$ implies that the difference between ETFS and SFSS is statistically significant

**Fig. 7:**
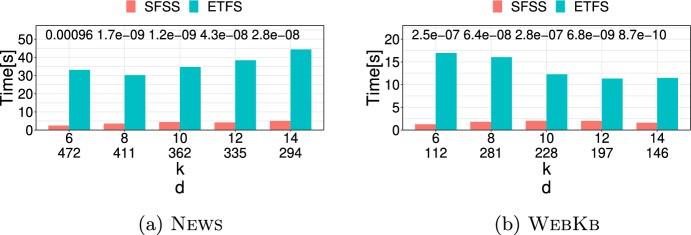
Runtime (secs), for varying length *k* of forbidden patterns, for:** a** News and** b** WebKb. The runtime values, as well as the number *d* of occurrences of forbidden patterns, are averages over 10 runs. On the top of each pair of bars, we plot the *p*-value of a *t*-test; $p< 0.05$ implies that the difference between ETFS and SFSS is statistically significant

However, SFSS is much faster than ETFS (e.g., 5 times faster on average in the News dataset). This can be seen in Figs. 6 and 7, which report the runtimes of these two sanitization methods. The result is consistent with the time complexities of the two algorithms: quadratic in the length of the input string for ETFS and linear in the length of the input string for SFSS (see Table 1). The differences in the runtimes of the two methods are statistically significant.

To summarize, the results in Figs. 4, 5, 6, and 7 are very encouraging because SFSS: (I) is equally effective to (and sometimes more effective than) ETFS despite offering better privacy, as it replaces the $\#$’s, unlike ETFS which does not and thus risks revealing the location of sensitive patterns; and (II) is also considerably faster (up to more than one order of magnitude) than ETFS.

### Large antidictionary

In this section, we evaluate the effectiveness and efficiency of our SFSS algorithm when the size of the antidictionary grows.

**Fig. 8:**
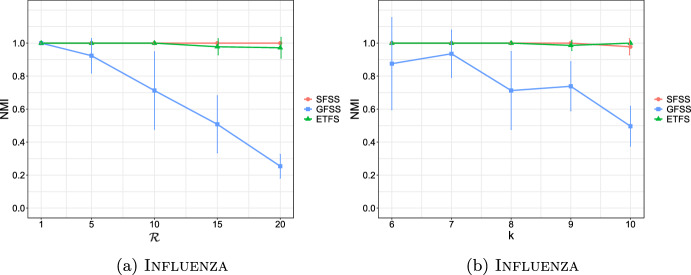
NMI for Influenza and varying:** a** $\mathcal {R}=\frac{|S_k|}{|\Sigma ^k|}\cdot 100\%$ and $k=8$, and** b** *k* and $\mathcal {R}=10\%$. The error bars are the corresponding standard deviations

**Fig. 9:**
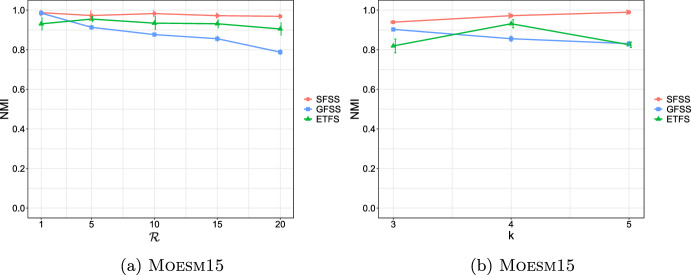
NMI for Moesm15 and varying:** a** $\mathcal {R}=\frac{|S_k|}{|\Sigma ^k|}\cdot 100\%$ and $k=4$, and** b** *k* and $\mathcal {R}=15\%$. The error bars are the corresponding standard deviations

**Fig. 10:**
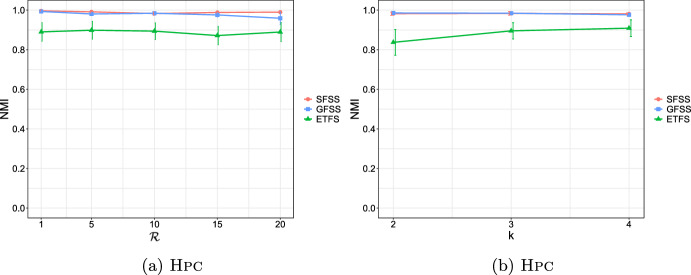
NMI for Hpc and varying:** a** $\mathcal {R}=\frac{|S_k|}{|\Sigma ^k|}\cdot 100\%$ and $k=3$, and** b** *k* and $\mathcal {R}=10\%$. The error bars are the corresponding standard deviations

***Real Datasets*** Figs. 8, 9, and 10 show that SFSS preserves clustering quality substantially better than GFSS and similarly or better than ETFS, according to the NMI measure; the results for ARI are analogous (omitted). The fact that these measures are 1 or close to 1 for SFSS demonstrates that it incurs no or insignificant clustering quality loss.

On the other hand, GFSS is often much worse than SFSS, achieving NMI scores as low as 0.25. Note that GFSS performed worse, heavily distorting the clustering structure of the data, when: (1) the alphabet size was small; (2) there were many occurrences of forbidden patterns; or (3) the forbidden patterns were long (i.e., *k* was large). For example, compare Fig. 8 with Fig. 10, in which the alphabet size is 4 and 65, respectively. GFSS performed much worse in the former case, whereas SFSS always achieved a near-perfect result. Also, observe in Fig. 8a that GFSS performed very poorly when a large fraction of length-*k* substrings were forbidden. This fraction is given by $\mathcal {R}=\frac{|S_k|}{|\Sigma ^k|}$. Similarly, GFSS performed poorly in Fig. 8b when *k* increased. The reason for this behavior is that deleting a letter removes an occurrence of a forbidden pattern $s\in S_k$ but may create an occurrence of another forbidden pattern $s'\in S_k$, or even another occurrence of *s*. Clearly, this is more likely to happen in cases 1, 2, 3 above. As an example, consider $w=\texttt {CAAAAAC}$, $k=3$, and $s=\texttt {AAA}$. GFSS will remove the third $\texttt {A}$ to prevent the first occurrence of *s* but this creates another occurrence of it starting at the beginning of the string. Thus, finally, GFSS will delete all $\texttt {A}$’s but the first two, constructing a sanitized string $y=\texttt {CAAC}$. On the other hand, SFSS would create a sanitized string $y'=\texttt {CAACAAC}$.

ETFS performed similarly to (and sometimes slightly worse than) SFSS, which is consistent with the experiments of Sect. 7.3. This can be seen in Figs. 8, 9, and 10. Also, unlike GFSS, its performance was not affected by the alphabet size, the number of occurrences of forbidden patterns, or the length of forbidden patterns.

**Fig. 11:**
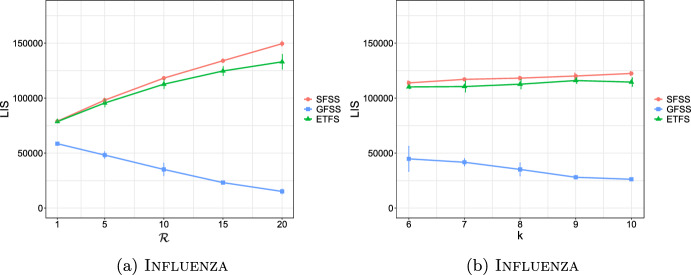
$\text {LIS}$ for Influenza and varying:** a** $\mathcal {R}=\frac{|S_k|}{|\Sigma ^k|}\cdot 100\%$ and $k=8$, and** b** *k* and $\mathcal {R}=10\%$. The error bars are the corresponding standard deviations

**Fig. 12:**
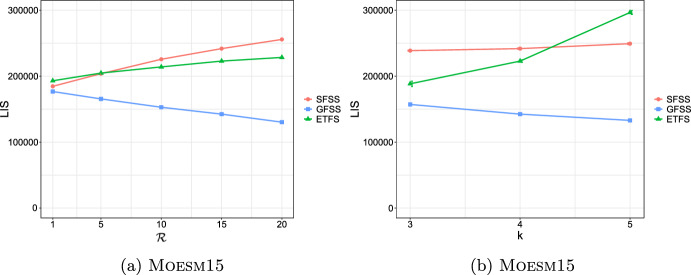
$\text {LIS}$ for Moesm15 and varying:** a** $\mathcal {R}=\frac{|S_k|}{|\Sigma ^k|}\cdot 100\%$ and $k=4$, and** b** *k* and $\mathcal {R}=15\%$. The error bars are the corresponding standard deviations

**Fig. 13:**
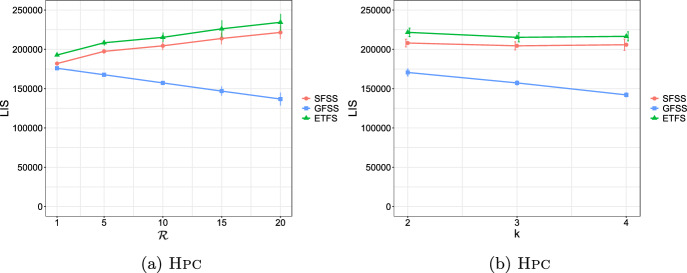
$\text {LIS}$ for Hpc and varying:** a** $\mathcal {R}=\frac{|S_k|}{|\Sigma ^k|}\cdot 100\%$ and $k=3$, and** b** *k* and $\mathcal {R}=10\%$. The error bars are the corresponding standard deviations

We also report the results of the experiments in Figs. 8, 9, and 10 with respect to the $\text {LIS}$ measure (see Sect. 7.2 for its definition) in Figs. 11, 12, and 13, respectively. Clearly, $\text {LIS}$ differs from the NMI measure in that it does not consider the clustering of the original data when capturing clustering quality. Instead, it considers how “compact” are the clusters of the sanitized data, treating a cluster as compact when its strings are similar to their cluster representative with respect to $\text {LIS}$. As can be seen, the results are analogous to those when NMI was used. That is, GFSS again performed much worse than SFSS, especially when the alphabet size was small, there were many occurrences of forbidden patterns, and *k* was large. On the other hand, ETFS performed much better than SFSS, being again comparable to SFSS.

**Fig. 14:**
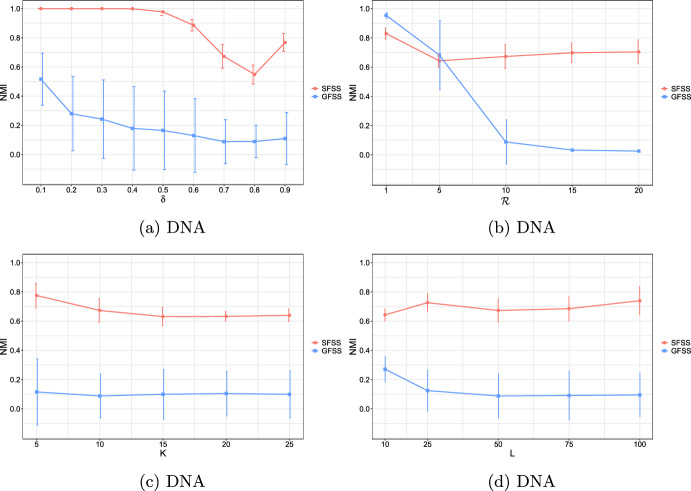
NMI for synthetic datasets constructed from a DNA dataset and varying:** a** $\delta$,** b** $\mathcal {R}$,** c** *K*, and** d** *L*. Default values are $k=8$, $\delta =0.7$, $\mathcal {R}=10\%$, $K=10$, and $L=50$. The error bars are the corresponding standard deviations

**Fig. 15:**
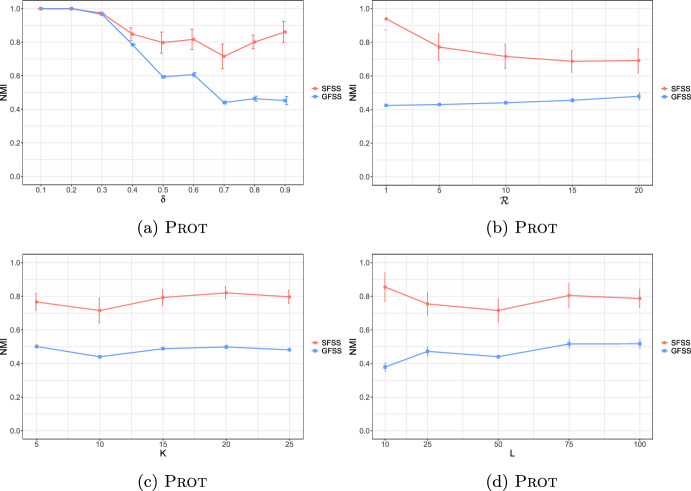
NMI for synthetic datasets constructed from a protein dataset and varying:** a** $\delta$,** b** $\mathcal {R}$,** c** *K*, and** d** *L*. Default values are $k=4$, $\delta =0.7$, $\mathcal {R}=10\%$, $K=10$, and $L=50$. The error bars are the corresponding standard deviations

***Synthetic Datasets*** We examine the impact of different parameters that affected the clustering quality of GFSS in the experiments of the paragraph “Real Data” above using synthetic data. We show in Figs. 14 and 15 that again GFSS performed substantially worse than SFSS in terms of being able to preserve clustering quality. Note that the NMI scores in the synthetic datasets used in Figs. 14 and 15 are generally lower than those obtained in the case of real datasets, used in the experiments above (the results for ARI are analogous (omitted)). This suggests that the synthetic datasets are more challenging to deal with. Yet, the results for SFSS are still very good, as the NMI scores were 0.78 on average, over all experiments of Figs. 14 and 15. As expected, the synthetic datasets generated with a larger *d* (number of occurrences of forbidden patterns) are more difficult to cluster, since their clusters are comprised of less similar strings. Thus, the NMI scores for both algorithms were in general lower for large values of *d* in Figs. 14a and 15a. Again, GFSS performed worse when the alphabet size was smaller (recall that the datasets in Fig. 14a have alphabet size 4 while those in Fig. 14a have alphabet size 20) and when the percentage of possible length-*k* substrings that are forbidden was larger (the effect of this was more evident in Fig. 14b due to the small alphabet size). It can be also noted in Figs. 14c, d, 15c, and d that our algorithm outperformed GFSS for all tested values of *K* (i.e., number of clusters) and *L* (i.e., number of strings in each cluster).

To summarize, the results in Figs. 8, 9, 10, 14, and 15 demonstrate that SFSS is able to preserve clustering quality. This is because it constructs clusterings that are identical or very similar to the clusterings of real datasets, or very similar to the clusterings of synthetic datasets, even though the synthetic datasets are constructed based on edit distance that is not optimized by SFSS.

### Scalability of SFSS

We examined the runtime of SFSS using the Chr and Prot datasets. We did not compare SFSS to ETFS, since ETFS did not scale to the size of these datasets, due to its quadratic complexity in |*w*|, the input string length (see Table 1). In addition, we omit the results of GFSS, since it was much faster albeit significantly less effective. This is because, as can be seen in Table 1, the term $k|S_k|$ in the time complexity of GFSS is replaced by $d\cdot k|S_k|\cdot |\Sigma |$ in the time complexity of SFSS (recall that *d* denotes the total number of occurrences of forbidden patterns in *w* and is bounded by |*w*|). Note that below we report the runtime just for the sanitization routine.

**Fig. 16:**
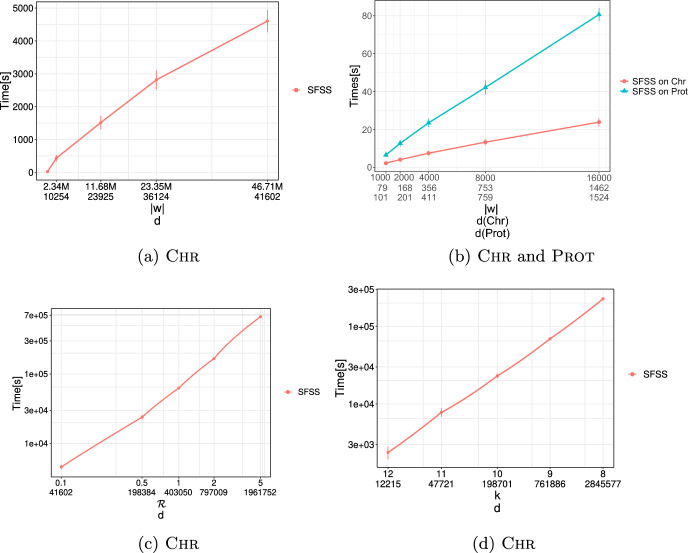
Runtime for varying:** a** |*w*| for Chr ($k=8$ and $\mathcal {R}=0.1\%$),** b** |*w*| for Chr and Prot (for Chr $k=8$, $\mathcal {R}=10\%$, $|\Sigma |=4$ and for Prot $k=4$, $\mathcal {R}=10\%$, $|\Sigma |=20$),** c** $\mathcal {R}=\frac{|\mathcal {S}_k|}{|\Sigma ^k|}\cdot 100\%$ for Chr ($k=8$), and** d** *k* for Chr ($\mathcal {R}=0.1\%$). The *x* axis of (a) to (d) also shows the total number of occurrences of forbidden patterns *d*. The error bars are the corresponding standard deviations

***Impact of*** |*w*| Fig. 16a shows that the runtime of SFSS grows linearly with respect to the input string length |*w*|, as expected by its time complexity. We also show that it is reasonably fast to be applicable to very long strings (recall that the Chr dataset has length of around 47 million letters).

***Impact of*** $|\Sigma |$ Fig. 16b shows that SFSS is slower in the case of the Prot dataset compared to the case of the Chr dataset, which has a larger alphabet size $|\Sigma |$. This experiment was performed using substrings of the Chr and Prot datasets and using $\mathcal {R}=10\%$ for both datasets. This result is in line with the time complexity of SFSS. Note that the larger runtime in the case of Prot is not only due to its larger alphabet size but also due to the much larger number *d* of occurrences of forbidden patterns, which is also a multiplicative factor in the time complexity of SFSS.

***Impact of*** $\mathcal {R}$ **and** *d* Fig. 16c shows that SFSS scales linearly with $\mathcal {R}=\frac{|S_k|}{|\Sigma ^k|}$. This experiment was performed using the entire Chr dataset and $k=8$. Note that by increasing $\mathcal {R}$, *d* also increases as the number of occurrences of forbidden patterns increases as well. Since $|\Sigma ^k|$ is fixed, the results in Fig. 16c imply that it scales linearly with the number $|S_k|$ of forbidden patterns, as expected by its time complexity. These results also imply that the runtime is linear in *d*, which changes as shown in the figures, as expected.

***Impact of*** *k* Fig. 16d shows the runtime of SFSS, for varying *k*. This experiment was performed using the entire Chr dataset and a fixed number $|S_k|=5000$ of forbidden patterns. The runtime of SFSS increases as *k* decreases, because the number *d* of occurrences of forbidden patterns increases. As *d* is significantly larger than *k*, its increase affects the runtime more than the decrease of *k*.

### Existing missing value replacement methods are not alternative to SFSS

We demonstrate that the well-established missing value replacement methods MIMR (Halpin 2016, 2012, 2013) and MissForest (Stekhoven and Bühlmann 2012) are *not* suitable to be used as alternatives to our SFSS algorithm. In particular, we demonstrate that they are unable to construct *feasible* (let aside *optimal*) solutions to the SFSS problem, as the strings they construct still contain a large number of forbidden patterns.

To show this, we used each of the aforementioned missing value replacement methods as a basis for two heuristics. The first heuristic is based on the idea of GFSS; the only difference is that, instead of deleting a letter *w*[*i*], the heuristic replaces *w*[*i*] with a letter output by one of the missing value replacement methods. The second heuristic uses the output of TFS as its input, as our SFSS algorithm does. However, different from SFSS, this heuristic employs missing value replacement methods to replace the $\#$’s in the output of TFS.

**Fig. 17:**
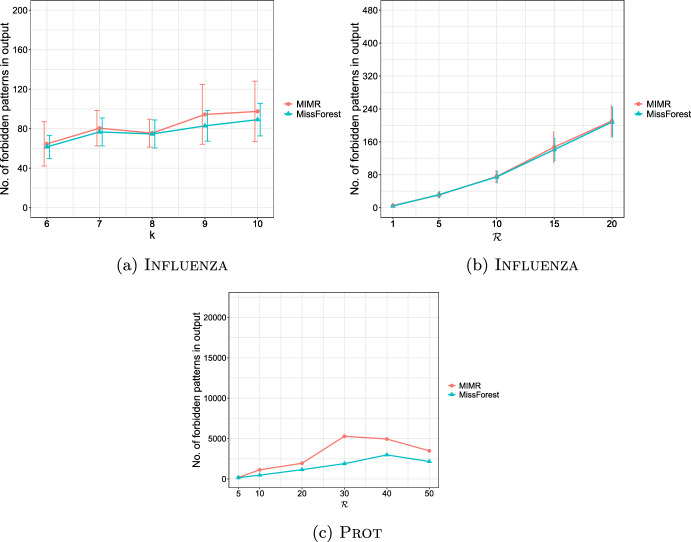
Number of forbidden patterns in the output of missing value replacement methods, MIMR (Halpin 2016, 2012, 2013) and MissForest  (Stekhoven and Bühlmann 2012), when coupled with the first heuristic, for varying:** a** *k* for Influenza ($$\mathcal {R}=\frac{|S_k|}{|\Sigma ^k|}\cdot 100\%=10\%$$),** b** $$\mathcal {R}$$ for Influenza ($$k=8$$), and** c** $$\mathcal {R}$$ for Prot ($$k=4)$$. The results in each *k* and $$\mathcal {R}$$ in (a) and (b) are averaged over all strings in Influenza and the error bars are the corresponding standard deviations

**Fig. 18:**
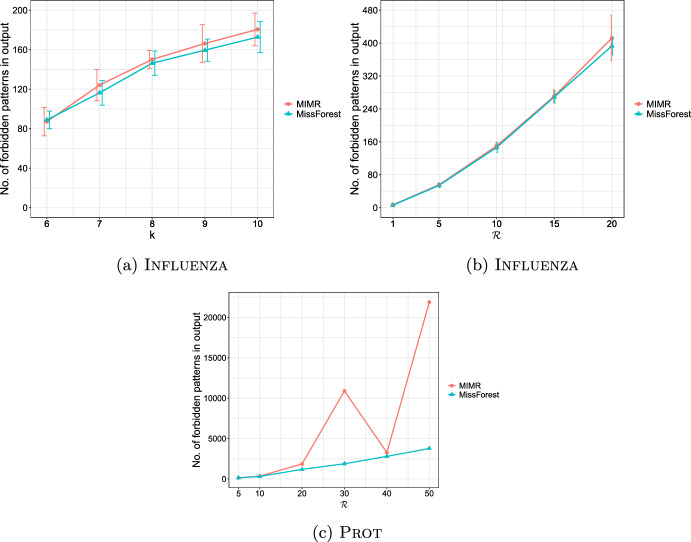
Number of forbidden patterns in the output of missing value replacement methods, MIMR (Halpin 2016, 2012, 2013) and MissForest  (Stekhoven and Bühlmann 2012), when coupled with the second heuristic, for varying:** a** *k* for Influenza ($$\mathcal {R}=\frac{|S_k|}{|\Sigma ^k|}\cdot 100\%=10\%$$),** b** $$\mathcal {R}$$ for Influenza ($$k=8$$), and** c** $$\mathcal {R}$$ for Prot ($$k=4)$$. The results in each *k* and $$\mathcal {R}$$ in (a) and (b) are averaged over all strings in Influenza and the error bars are the corresponding standard deviations

We start by examining the effectiveness of the first heuristic. Figure 17a shows that, when applied to the Influenza dataset, the heuristic produces outputs containing forbidden patterns, for all tested values of *k*, irrespectively of whether it is coupled with MIMR or with MissForest. This implies that neither MIMR nor MissForest is appropriate to be used as an alternative to our SFSS algorithm, which guarantees that no forbidden patterns will be present in its output. Furthermore, the number of forbidden patterns contained in the output of either version of the heuristic increases with *k*. This is because there are more forbidden patterns in the input string when *k* is larger and thus there is a larger chance that some of them are retained in the output. The results shown in Fig. 17b are analogous: there are always forbidden patterns in the output of either version of the heuristic, for all tested values of $\mathcal {R}$. The number of these patterns increases with $\mathcal {R}$, as more forbidden pattern occurrences in the input are candidates for being retained in the output when $\mathcal {R}$ is larger. In addition to Influenza, which is a collection of strings, we used Prot, which is a single, longer string. The results in Fig. 17c are analogous to those in Fig. 17b: there is a large number of forbidden patterns in the output of either version of the heuristic, which increases with $\mathcal {R}$.

We now examine the effectiveness of the second heuristic. Figure 18a shows that, when applied to the Influenza dataset, the heuristic produces outputs containing forbidden patterns, for all tested values of *k*, irrespectively of whether it is coupled with MIMR or MissForest. The same happens in the results of Fig. 18b, which are produced by varying $\mathcal {R}$. Again, the number of forbidden patterns in the output increases with *k* and/or $\mathcal {R}$, as there are more forbidden patterns in the input that can possibly be retained in the output of the heuristic. Figure 18c shows the results for the Prot dataset, which are analogous to those in Fig. 17c.

Last, by comparing Fig. 18 to Fig. 17, one can notice that the second heuristic is in general less effective than the first one, but none of them was able to produce a string that contains no forbidden patterns in any tested case. This is in sharp contrast to our SFSS algorithm, which not only guarantees that no forbidden pattern occurs in output but it also enjoys other useful properties. As demonstrated in Sect. 7, these properties play a crucial role in preserving clustering quality.

## Conclusion and future work

Missing value replacement in strings is an important task, as strings with missing values are encountered in many applications, ranging from bioinformatics to data sanitization and databases.

In this paper, we formalized the task of missing value replacement in strings as a combinatorial optimization problem. Our formulation considers the context of a missing value to preserve the sequential nature of the string, as well as a set of forbidden patterns to avoid introducing spurious or confidential information; and seeks to minimize the information added by the replacement.

We designed an algorithm that solves this problem in linear time for strings over constant-sized alphabets. We also proposed a methodology for sanitizing and clustering a collection of private strings that utilizes our algorithm as well as an effective and efficiently computable distance measure. Last, we presented extensive experiments demonstrating that our methodology can sanitize a collection of private strings while preserving clustering quality outperforming the state of the art and a greedy baseline. We leave the following directions open for future investigation:

1. The main open question is: Can the MVRS problem be solved faster than $\mathcal {O}(|u|+|v|+||S||\cdot |\Sigma |)$ time? One should perhaps design a fundamentally different technique that avoids the DFA construction, because, as we have shown, the latter has $\Omega (||S||)$ states and $\Omega (||S||\cdot |\Sigma |)$ edges.
2. The work of (Bernardini et al 2021b) investigated whether the decision version of MVRS for forbidden patterns of fixed length *k* can be solved faster than $\mathcal {O}(k|S_k|\cdot |\Sigma |)$ time. Formally, given an integer $k>0$, two strings $u,v\in \Sigma ^{k-1}$, and a set $S_k\subset \Sigma ^{k}$, the question is: Does there exist a string $x\in \Sigma ^{*}$ such that *u* is a prefix of *x*, *v* is a suffix of *x*, and no $s\in S_{k}$ occurs in *x*? This work also proposed a non-constructive randomized algorithm that solves this problem in the optimal $\mathcal {O}(k|S_k|)$ time. With *non-constructive* we mean that no witness string *x* is output. This may be useful to check fast whether or not we can solve an instance of the MVRS problem, and if not, change the input. If the answer is YES, one would want to output a (shortest) witness string as well, which can be done in $\mathcal {O}(k|S_k|\cdot |\Sigma |)$ time using our algorithm for MVRS. The open question is: Can this decision version of the MVRS problem be solved deterministically in $\mathcal {O}(k|S_k|)$ time?
3. Sometimes we may want to solve many instances of the MVRS problem having the same set *S* of forbidden patterns but different *u* and *v*; for example, in the SFSS problem. The open question is: Can we solve *q* such instances faster than applying Theorem 1*q* times?
4. In the definition of the MVRS problem, we assume that the set *S* of forbidden patterns is finite; however the MVRS problem may be generalized to the case where *S* is a regular language, i.e., the constraints (forbidden patterns) arise from a set of regular expressions. It might be interesting to investigate this problem both from the theory and the practical perspective.

## Footnotes

1 We give a formal definition of the *k*-gram distance (Ukkonen 1992). Let *G*(*x*)[*v*] denote the total number of occurrences of string *v* in string *x*. Fix an enumeration order to list all possible *k*-grams (i.e., length-*k* strings) over $\Sigma$. The *k*-*gram profile* of *x* is the vector $G_k(x)=(G(x)[v]),v\in \Sigma ^k$. The *k*-*gram distance* between two strings is defined as the $L_1$-norm of the difference of their two corresponding *k*-gram profiles.

2 In graph theory, reachability refers to the ability to get from node *u* to node *v* in a given graph.

3 In what follows, we use the term graph and automaton interchangeably, depending on the context.

4 We can run two DFAs simultaneously on the same input via the so-called *product construction.*

5 Note that by “sequence of *k*-grams” we mean a sequence of *k*-gram *items* rather than a concatenation of strings; therefore, by “subsequence” we mean a subsequence of these items, rather than a subsequence of their letters. For instance, the sequence of 2-grams of $u=\texttt {aabaabb}$ is $\langle \texttt {aa,ab,ba,aa,ab,bb} \rangle$; $\langle \texttt {aa,ab,bb} \rangle$ is a valid subsequence, whereas $\langle \texttt {aa,bb,ab} \rangle$ is not, because the 2-gram bb appears in *u* only after ab.

6 Note that the term data sanitization has sometimes been used more generally as a synonym of privacy-preserving data publishing; see e.g.,  (Li et al 2017a; Hong et al 2012). We use it precisely as explained above.

7 A ghost pattern is a length-*k* substring that occurs infrequently in the string to be sanitized (i.e., fewer times than a given threshold) but frequently in the sanitized string.

8 This was performed by sorting the words lexicographically, transforming them into integers based on their rank and mapping the same number of consecutive integers to an integer in [1, 100]. This operation made the datasets less sparse (their strings became more similar), so that it is possible to construct reasonably coherent clusters. A smaller $|\Sigma |$ also improves the efficiency of our algorithm.

9 This was a length-27881 string that was much longer than all others.
